# Anticancer chemotherapy and radiotherapy trigger both non-cell-autonomous and cell-autonomous death

**DOI:** 10.1038/s41419-018-0747-y

**Published:** 2018-06-18

**Authors:** Isabelle Martins, Syed Qasim Raza, Laurent Voisin, Haithem Dakhli, Awatef Allouch, Frédéric Law, Dora Sabino, Dorine De Jong, Maxime Thoreau, Elodie Mintet, Delphine Dugué, Mauro Piacentini, Marie-Lise Gougeon, Fanny Jaulin, Pascale Bertrand, Catherine Brenner, David M. Ojcius, Guido Kroemer, Nazanine Modjtahedi, Eric Deutsch, Jean-Luc Perfettini

**Affiliations:** 1 Cell Death and Aging Team, Gustave Roussy Cancer Campus, 114 rue Edouard Vaillant, F-94805 Villejuif, France; 2 Laboratory of Molecular Radiotherapy, INSERM U1030, Gustave Roussy Cancer Campus, 114 rue Edouard Vaillant, F-94805 Villejuif, France; 3 Gustave Roussy Cancer Campus, 114 rue Edouard Vaillant, F-94805 Villejuif, France; 4 Université Paris Saclay, 114 rue Edouard Vaillant, F-94805 Villejuif, France; 5 Institute of Biochemistry and Biotechnology, University of Veterinary and Animal Sciences, UVAS Syed Abdul Qadir Jilani (Out Fall) Road, Lahore, 54200 Pakistan; 6 INSERM U981, Gustave Roussy Cancer Campus, 114 rue Edouard Vaillant, F-94805 Villejuif, France; 7 National Institute for Infectious Diseases “Lazzaro Spallanzani”, Via Portuense 292, 00149 Rome, Italy; 8 Department of Biology, University of Rome “Tor Vergata”, Via della Ricerca Scientifica 1, 00133 Rome, Italy; 9 Antiviral Immunity, Biotherapy and Vaccine Unit, Institut Pasteur, 25 rue du Dr. Roux, F-75015 Paris, France; 10 DNA Repair and Aging Team, Commissariat à l’Energie Atomique, Direction Recherche Fondamentale, Institut de Biologie François Jacob, INSERM UMR 967 CEA, Université Paris Diderot, Université Paris Saclay, 18, route du Panorama, F-92265 Fontenay-aux-Roses, France; 11 Laboratory of Signalling and Cardiovascular Pathophysiology, INSERM UMR-S 1180, Université Paris-Sud, Université Paris Saclay, 5 rue Jean-Baptiste Clément, F-92296 Châtenay-Malabry, France; 12 Department of Biomedical Sciences, University of the Pacific, School of Dentistry, 155 Fifth Street, San Francisco CA, 94103 USA; 13 Equipe 11 Labellisée par la Ligue Contre le Cancer, Centre de Recherche des Cordeliers, Paris, France; 14 Cell Biology and Metabolomics Platforms, Gustave Roussy Cancer Campus, Villejuif, France; 15 Université Paris Descartes, Sorbonne Paris Cité, Paris, France; 16 Université Pierre et Marie Curie, Paris, France; 17 Pôle de Biologie, Hôpital Européen Georges Pompidou, AP-HP, Paris, France; 18 Karolinska Institute, Department of Women’s and Children’s Health, Karolinska University Hospital, Stockholm, Sweden

## Abstract

Even though cell death modalities elicited by anticancer chemotherapy and radiotherapy have been extensively studied, the ability of anticancer treatments to induce non-cell-autonomous death has never been investigated. By means of multispectral imaging flow-cytometry-based technology, we analyzed the lethal fate of cancer cells that were treated with conventional anticancer agents and co-cultured with untreated cells, observing that anticancer agents can simultaneously trigger cell-autonomous and non-cell-autonomous death in treated and untreated cells. After ionizing radiation, oxaliplatin, or cisplatin treatment, fractions of treated cancer cell populations were eliminated through cell-autonomous death mechanisms, while other fractions of the treated cancer cells engulfed and killed neighboring cells through non-cell-autonomous processes, including cellular cannibalism. Under conditions of treatment with paclitaxel, non-cell-autonomous and cell-autonomous death were both detected in the treated cell population, while untreated neighboring cells exhibited features of apoptotic demise. The transcriptional activity of p53 tumor-suppressor protein contributed to the execution of cell-autonomous death, yet failed to affect the non-cell-autonomous death by cannibalism for the majority of tested anticancer agents, indicating that the induction of non-cell-autonomous death can occur under conditions in which cell-autonomous death was impaired. Altogether, these results reveal that chemotherapy and radiotherapy can induce both non-cell-autonomous and cell-autonomous death of cancer cells, highlighting the heterogeneity of cell death responses to anticancer treatments and the unsuspected potential contribution of non-cell-autonomous death to the global effects of anticancer treatment.

## Introduction

From the initial discovery of programmed cell death during animal development^1^ to the recent identification of entotic death during embryo implantation^2^, a cornucopia of cell death modalities has been identified and shown to play a role in numerous physiological or pathological situations^3, 4^. Mainly studied as clonal cellular responses to lethal stress, cell death processes have been defined on the basis of their specific morphological features (e.g., apoptotic, autophagic, or necrotic), their metabolic and biochemical characteristics (e.g., the loss of mitochondrial transmembrane potential, the exposure of phosphatidylserine (PS) on the outer leaflet side, or the rupture of plasma membrane integrity), their enzymatic and catabolic activities (involving (or not) caspases, receptor-interacting protein kinases (RIPKs), mixed lineage kinase domain-like proteins, or cathepsins), and in relation to their ability to elicit an inflammatory reaction or to stimulate an immune response. A classification of cell death modalities built on these criteria has been proposed^5^ and led to the ordering of lethal processes into three distinct types: type I cell death (or apoptosis), type II cell death (or autophagic cell death), and type III cell death (or necrosis). All these processes, which are executed in a cell-autonomous manner, can be induced in the targeted stressed cells or at a distance, in the neighboring cells (through bystander effects). These processes are known as cell-autonomous death (CAD)^6^. Despite major progresses that have been made in the field, the relative contribution of both direct and bystander-signal-mediated killing triggered by typical CAD remains poorly explored.

Cell death subroutines (such as mitotic death and cornification) that do not or partially exhibit the typical morphological and biochemical hallmarks of cell death have been less studied and are listed in a poorly defined subgroup of cell death modalities known as atypical cell death^5^. In recent years, additional cell death mechanisms (such as entosis or emperitosis) have been described and associated with this neglected subgroup of cell death modalities^7, 8^. Their examination revealed the existence of cell death processes that are elicited after the engulfment of live cells by neighboring live cells. These lethal processes are also known as non-cell-autonomous death (NCAD). The first step of NCAD programs, which start with the interaction of two cellular partners through membrane adhesion receptors (such as E- or P-cadherins) or stress receptors (such as lipoprotein receptor-related protein), requires the formation of adherent junctions between interacting cells and the activation of signaling pathways, which may involve small GTPases (such as Rho^9^ and cell division cycle 42 (CDC42)^10^) and ROCK kinases^7^, on both interacting cells. The modulation of actomyosin contractility and the reorganization of the actin cytoskeleton in “target” cells also favor their invasion into host cells^9, 11^. This process is distinct from cellular cannibalism, which can also trigger NCAD through the activation of specific signaling pathways (such as phagocytosis-related signaling pathways that involve CDC42, chemokine (C-X-C motif) ligand 1 (CXCL1) or CXCL6) on host cells and leads to the active engulfment of target cells^10^. Independently of the precise cell engulfment process, engulfed cells are targeted by “host” lysosomal enzymes (such as cathepsins and granzymes) and eliminated through distinct lethal mechanisms that may involve major modulators of typical cell death (such as cytochrome *c*, caspases, or autophagy-related (ATG) proteins). When NCAD leads to the apoptotic demise of engulfed cells, the process is called phagoptosis^12–14^ or emperipolesis^15^. This form of cell death has been proposed to occur frequently in the body^12^ and to control erythrocyte, neutrophil, platelet, and T cell homeostasis^12, 16, 17^. The engulfment of natural killer cells by tumor cells was also shown to induce emperitosis, which is a programmed cell-in-cell death process that requires caspase-3 activation and leads to DNA fragmentation^8^. Inversely, entosis, another form of NCAD initially described after homotypic interactions between breast cancer cells, is a cell-in-cell invasion mechanism that does not require the activation of caspases to eliminate engulfed cells^7^. We recently defined NCAD as type IV cell death^6^.

Despite intensive biological and pharmaceutical research programs that have helped to better characterize cellular and biochemical processes associated with anticancer treatment, the ability of anticancer agents to simultaneously induce CAD and NCAD has never been investigated. Here, using novel multispectral imaging flow-cytometry-based methodology, we show that cancer cells respond to treatment with various anticancer agents by undergoing simultaneously several death modalities that can be either cell-autonomous or non-cell-autonomous and are distinctly impacted by the tumor-suppressive factor p53, revealing unsuspected heterogeneity of cell death responses to anticancer treatment.

## Results

### Ionizing radiation (IR) induces CAD of irradiated cancer cells

**Fig. 1:**
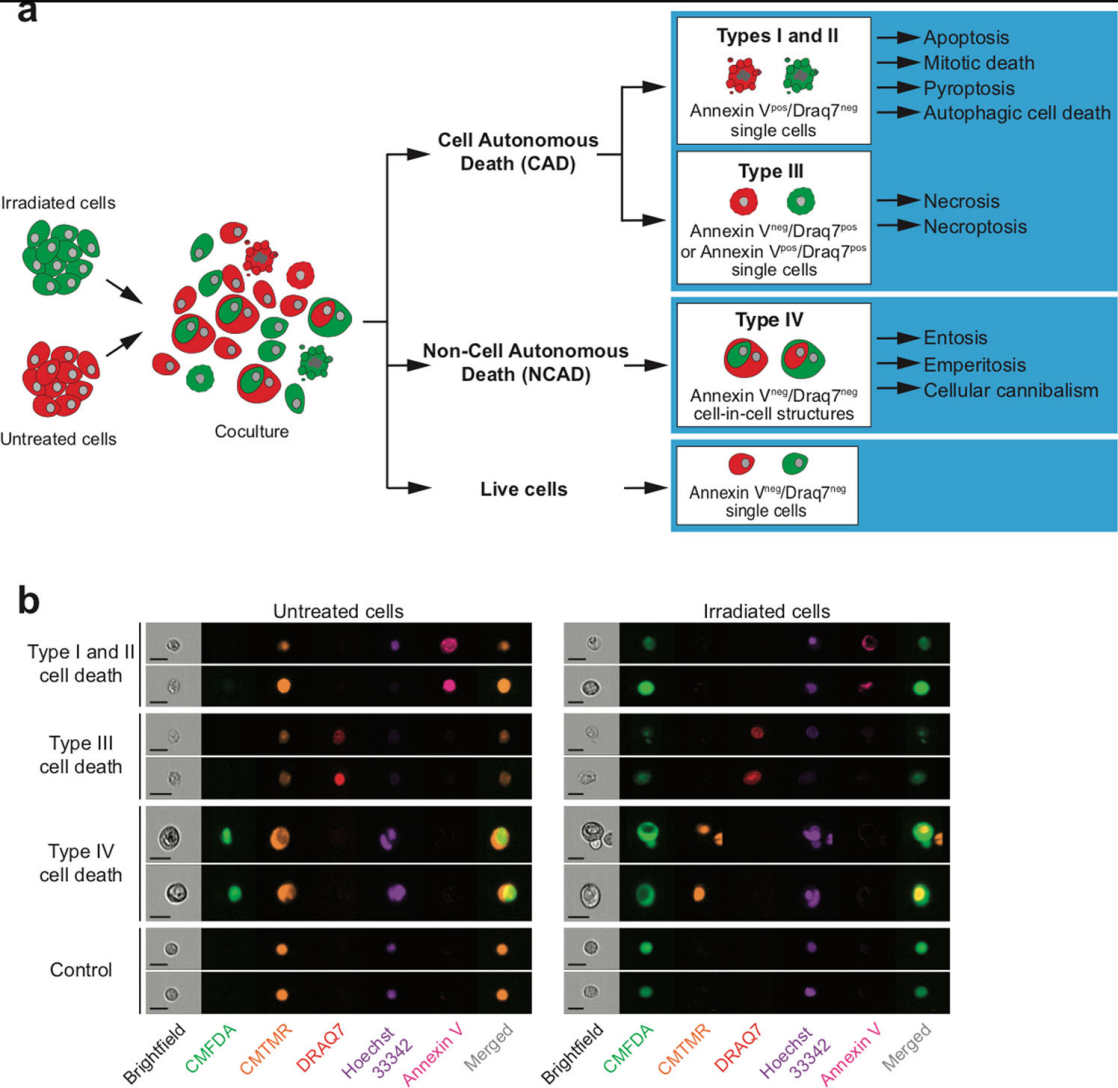
Detection of cell-autonomous and non-cell-autonomous death by confocal microscopy and quantitative imaging flow cytometry. **a** Principle of cell death profiling by quantitative flow imaging. Before co-culture, treated and non-treated cancer cells were, respectively, labeled with CMFDA (green) or CMTMR (red) fluorescent vital probes. After 24 h of co-culture, cancer cells were analyzed for non-cell-autonomous death (NCAD) (by detecting the engulfment of CMTMR- or CMFDA-labeled cancer cells), the phosphatidylserine (PS) exposure (using Biotin-AnnexinV and BV786-Streptavidin), the loss of plasma integrity (by following with DRAQ7 uptake), and the DNA content (using Hoechst 33342). Using quantitative flow cytometry, the simultaneous detection of NCA death (also known as type IV cell death) and of typical cell death (type I, II, and III) in both non-treated and treated cancer cell populations, the cell death profiling is determined for various anticancer treatments. **b** Validation of multiparametric and simultaneous detection of cell death modalities by quantitative imaging flow cytometry induced by γ-irradiation on human colon carcinoma HCT116 cells. Representative images are shown (scale, 20 μm)

Even though radiotherapy is one of the most frequent anticancer treatments used in the clinic, the lethal mechanisms responsible for the therapeutic effects of radiotherapy are still largely unknown. Lethal processes (such as apoptosis and mitotic catastrophe) that have been detected in response to IR were never directly implicated in treatment efficiency^18^, suggesting that additional, uncharacterized cell death modalities may contribute to the therapeutic effects of radiotherapy. To precisely analyze the cellular mechanisms through which cancer cells may simultaneously undergo direct and bystander cell killing in response to IR, we designed a novel cell death profiling assay based on co-culture of untreated cancer cells that have been labeled with the red fluorescent probe 5-(and-6)-(((4-chloromethyl)benzoyl)amino) tetramethylrhodamine (CMTMR) and irradiated isogenic cancer cells that have been labeled with the green fluorescent probe 5-chloromethylfluorescein diacetate (CMFDA). After different times of co-culture and exposure to different doses of IR, irradiated CMFDA^+^ cells, non-irradiated CMTMR^+^ cells, and the total cell population (CMFDA^+^ and CMTMR^+^) were analyzed for PS exposure, loss of plasma membrane integrity, and DNA content (Fig. 1a). The simultaneous detection of the above-indicated parameters allowed us to detect, through the use of multispectral imaging flow cytometry, the execution of at least four types of cell death (including several lethal pathways, such as apoptosis, mitotic death, pyroptosis, autophagic cell death, necrosis, necroptosis, entosis, emperitosis, and cellular cannibalism) on target cells and on neighboring cells (Fig. 1b), thus discriminating CAD from NCAD and direct cell killing from bystander lethal effects.

**Fig. 2:**
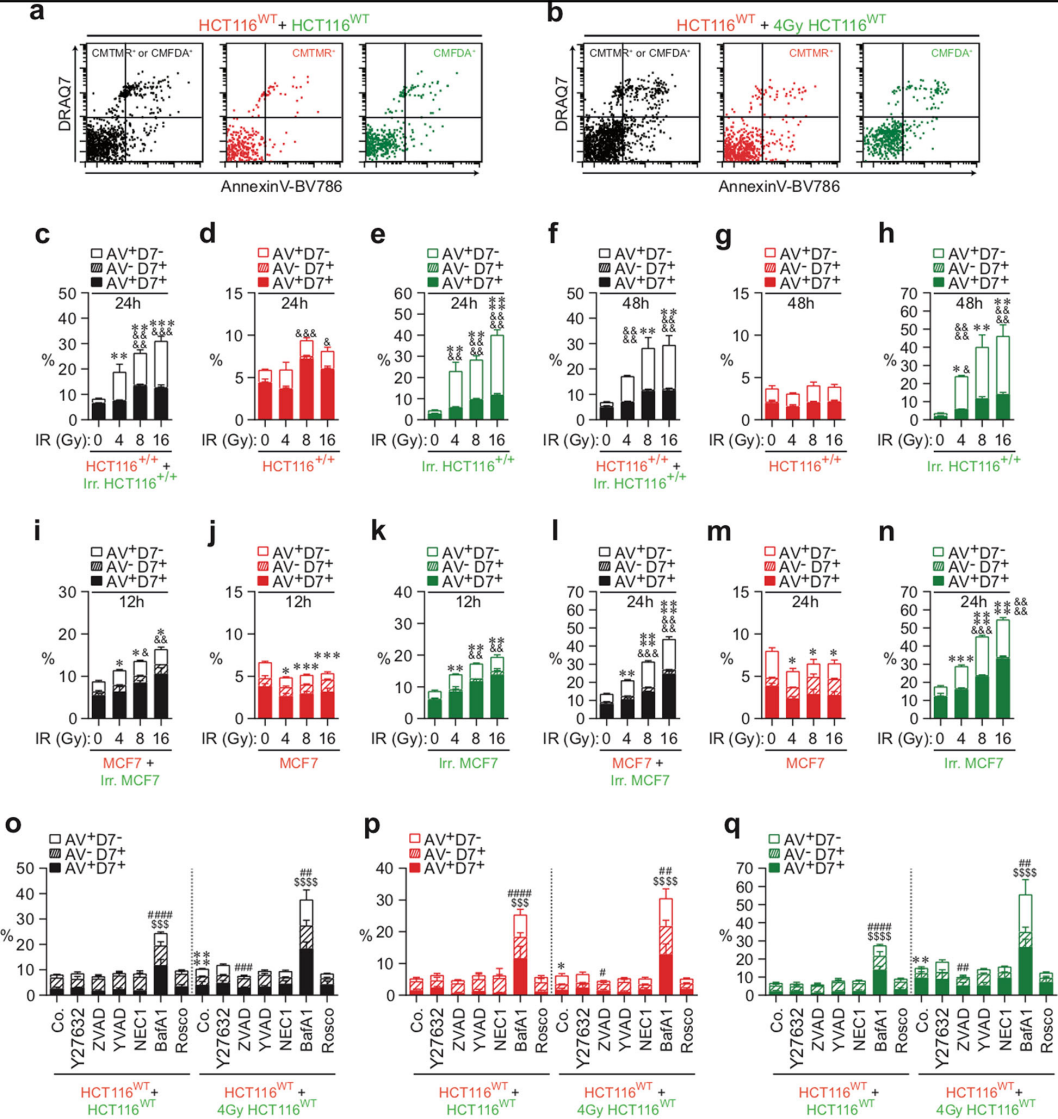
Detection of γ-irradiation–elicited CAD modalities by quantitative imaging flow cytometry. **a**–**h** Detection and quantification of plasma membrane integrity loss (with DRAQ7) and PS exposure (with BV786-streptavidin/Annexin V biotin) were achieved for total cell populations (CMTMR^+^ or CMFDA^+^ HCT116^WT^ cells (**a**, **o**), HCT116^+/+^ cells (**c**, **f**), and MCF7 cells (**i**, **l**)), for the untreated (red) CMTMR^+^ cell populations (HCT116^WT^ cells (**a**, **b**, **o–q**), HCT116^+/+^ cells (**c**, **d**, **f**, **g**), and MCF7 cells (**i**, **j**, **l**, **m**)), for untreated (green) CMFDA^+^ cell populations (HCT116^WT^ cells (**a**, **o**, **q**), HCT116^+/+^ cells (**c**, **e**, **f**, **h**), and MCF7 cells (**i**, **k**, **l**, **n**)), and for treated (green) CMFDA^+^ cell populations (HCT116^WT^ cells (**b**, **o**, **q**), HCT116^+/+^ cells (**c**, **e**, **f**, **h**), and MCF7 cells (**i**, **k**, **l**, **n**). CAD modalities were detected after 12-h (**i**–**k**), 24-h (**a**–**e**), or 48-h (**f**–**h**) co-culture of untreated (red) CMTMR-labeled cells with untreated (green) CMFDA-labeled cells (**a**, **c**, **f**, **i**, **l**) or of untreated (red) CMTMR-labeled cells with (green) CMFDA-labeled cells that have been irradiated with the indicated doses of γ-ionizing radiation (**b**, **d**, **e**, **g**, **j**, **k**, **m**, **n**). **o**–**q** Co-cultures of HCT116^WT^ cells were also performed during 24 h in the presence or absence of the indicated pharmacological death effector inhibitors. CAD modalities were determined as previously described for the untreated (red) CMTMR^+^ HCT116^WT^ cells, for untreated (green) CMFDA^+^ HCT116^WT^ cells, for treated (green) CMFDA^+^ HCT116^WT^ cells, and for total cell population (CMTMR^+^ or CMFDA^+^ HCT116^WT^ cells). Representative dot plots (**a**, **b**) and quantitative data (**c**–**q**) are shown (means ± SEM, *n* = 3). For  **c**–**h**, asterisk (*) is used for the comparison of “HCT116^+/+^+Irr. HCT116^+/+^” with “HCT116^+/+^+0 Gy HCT116^+/+^” for AV^+^D7^−^ and ampersand (&) is used for the comparison of “HCT116^+/+^+Irr. HCT116^+/+^” with “HCT116^+/+^+0 Gy HCT116^+/+^” for D7^+^. For **i**–**n**, asterisk (*) is used for the comparison of “MCF7+Irr. MCF7” with “MCF7+0 Gy MCF7” for AV^+^D7^−^ and ampersand (&) for the comparison of “MCF7+Irr. MCF7” with “MCF7+0 Gy MCF7” for D7^+^. For **o**–**q**, asterisk (*) is used for comparison of “HCT116^WT^+4 Gy control (Co.) HCT116^WT^” with “HCT116^WT^+control (Co.) HCT116^WT^” for AV^+^D7^−^, hash (#) for the comparison of inhibitor-treated cells with respective control cells for AV^+^D7^−^ and dollar symbol ($) for the comparison of inhibitor-treated cells with respective control cells for D7^+^. *^, #, &^*p* < 0.05; **^, ##, &&^*p* < 0.01; ***^, ###, $$$, &&&^*p* < 0.001; and ****^, ####, $$$$, &&&&^*p* < 0.0001

Thus CMFDA-labeled HCT116 human colon cancer cells (wild-type (WT) HCT116 (HCT116^WT^) cells, isogenic control HCT116 (HCT116^+/+^) cells, and HCT116^p53R248W/+^ cells harboring the dominant-negative mutant of p53, p53R248W (Fig. 2a–h), or MCF7 human breast cancer cells (Fig. 2i–n) were irradiated with different doses (0, 4, 8, and 16 Gy), mixed after 24 h with CMTMR-labeled isogenic cells (1:1 ratio), and cultured for the indicated times. PS exposure, plasma membrane integrity, and DNA content of each population were then determined by means of AnnexinV-BV786 (AV), DRAQ7 (D7), and Hoechst 33342 staining, respectively. Although no significant increase of apoptotic and necrotic cell death was observed in the untreated control cell population (as revealed by the detection of AnnexinV^+^DRAQ7^−^ (AV^+^D7^−^), AnnexinV^−^DRAQ7^+^ (AV^−^D7^+^), and AnnexinV^+^DRAQ7^+^ (AV^+^D7^+^) cells (Fig. 2a, c–h)), a significant increase of AV^+^D7^−^ cells and AV^+^D7^+^ cells was detected in the total population of HCT116^WT^ or HCT116^+/+^ cells (as revealed by CMFDA^+^ and CMTMR^+^ cells) (Fig. 2b, c, f, o), in neighboring HCT116^WT^ or HCT116^+/+^ cells (CMTMR^+^ cells) (Fig. 2b, d, g, p), and in irradiated HCT116^WT^ or HCT116^+/+^ cells (CMFDA^+^ cells) (Fig. 2b, e, h, q). These processes were observed in a dose-dependent manner after 24 h (Fig. 2c–e) and 48 h (Fig. 2f–h) of co-culture, demonstrating that our methodology allows us to score the death of both non-irradiated and irradiated cells. These results were confirmed after 12 h (Fig. 2i–k) and 24 h (Fig. 2l–n) of co-culture of control or irradiated CMFDA-labeled MCF7 cells with untreated CMTMR-labeled MCF7 cells. To characterize molecular mechanisms involved in the execution of these cell death processes, co-cultures of HCT116^WT^ cells were performed in the presence of cell death modulators (Supplementary Figures 1a-e). Thus the ROCK-1 inhibitor Y27632 was used as an inhibitor of live cell engulfment following procedures of previously published reports^7^. The peptide derivatives benzyloxycarbonyl-Val-Ala-Asp(OMe)-fluoromethylketone (Z-VAD-fmk) and YVAD-cmk were used as pan-caspase or caspase-1 inhibitors, respectively^19, 20^, and the RIPK1 inhibitor necrostatin-1 (NEC1) was employed as necroptosis inhibitor^21^. Bafilomycin A1 (BafA1) and the cyclin-dependent kinase inhibitor roscovitine (Rosco) were used to analyze the contribution of autophagic flux and mitotic progression, respectively^22–24^. We observed that the pan-caspase inhibitor Z-VAD-fmk reduced the exposure of PS on the plasma membrane of the total cell population of HCT116^WT^ cells (Fig. 2o), of irradiated CMFDA^+^ HCT116^WT^ cells (Fig. 2q), and to a lower extent, those of non-irradiated CMTMR^+^ HCT116^WT^ cells (Fig. 2p). These results confirm the notion that irradiated CMFDA^+^ cells die efficiently in the event of caspase activation^25^. However, co-culture experiments performed with MCF7 cells that are deficient for caspase-3 revealed that both total (Fig. 2i, l) and irradiated cell population (Fig. 2j, n) also exhibited a significant increase of AV^+^D7^−^ and AV^+^D7^+^ cells (as compared to control cells), thus indicating that irradiated cancer cells can also die through caspase-3-independent death. In addition, the impairment of autophagic flux with BafA1 increased the frequency of dying cells (AV^+^D7^−^, AV^+^D7^−^, and AV^+^D7^+^ cells) in the total cell population (CMFDA^+^ and CMTMR^+^ cells) (Fig. 2o), in non-irradiated CMTMR^+^ cells (Fig. 2p), and in irradiated CMFDA^+^ cells (Fig. 2q), pointing to autophagy as a pro-survival mechanism that contributes to rescue both non-irradiated and irradiated cells from death. The simultaneous analysis of the progression of non-irradiated and irradiated HCT116^WT^ cells (Supplementary Figures 2a, 2b, and 2o-2s), HCT116^+/+^ cells (Supplementary Figures 2c-2h), or MCF7 cells (Supplementary Figures 2i-2n) through their cell cycle compartment distribution showed that cell death induction was associated with the accumulation of irradiated cell populations in G2/M and 4N phases (Supplementary Figures 2a-2q). No alteration of the cell cycle was detected in the non-irradiated CMTMR^+^ cell populations (Supplementary Figures 2b, 2d, 2g, 2j, 2m, and 2p), implying that the cell cycle alterations were only detected in irradiated cells (Supplementary Figures 2b, 2e, 2h, 2k, 2n, and 2q). These results were confirmed by classical flow-cytometric analysis (Supplementary Figures 2r and 2s). Altogether, these results demonstrate that IR eliminates cancer cells mainly through direct cell killing.

### Paclitaxel induces CAD of both treated and neighboring cells

**Fig. 3:**
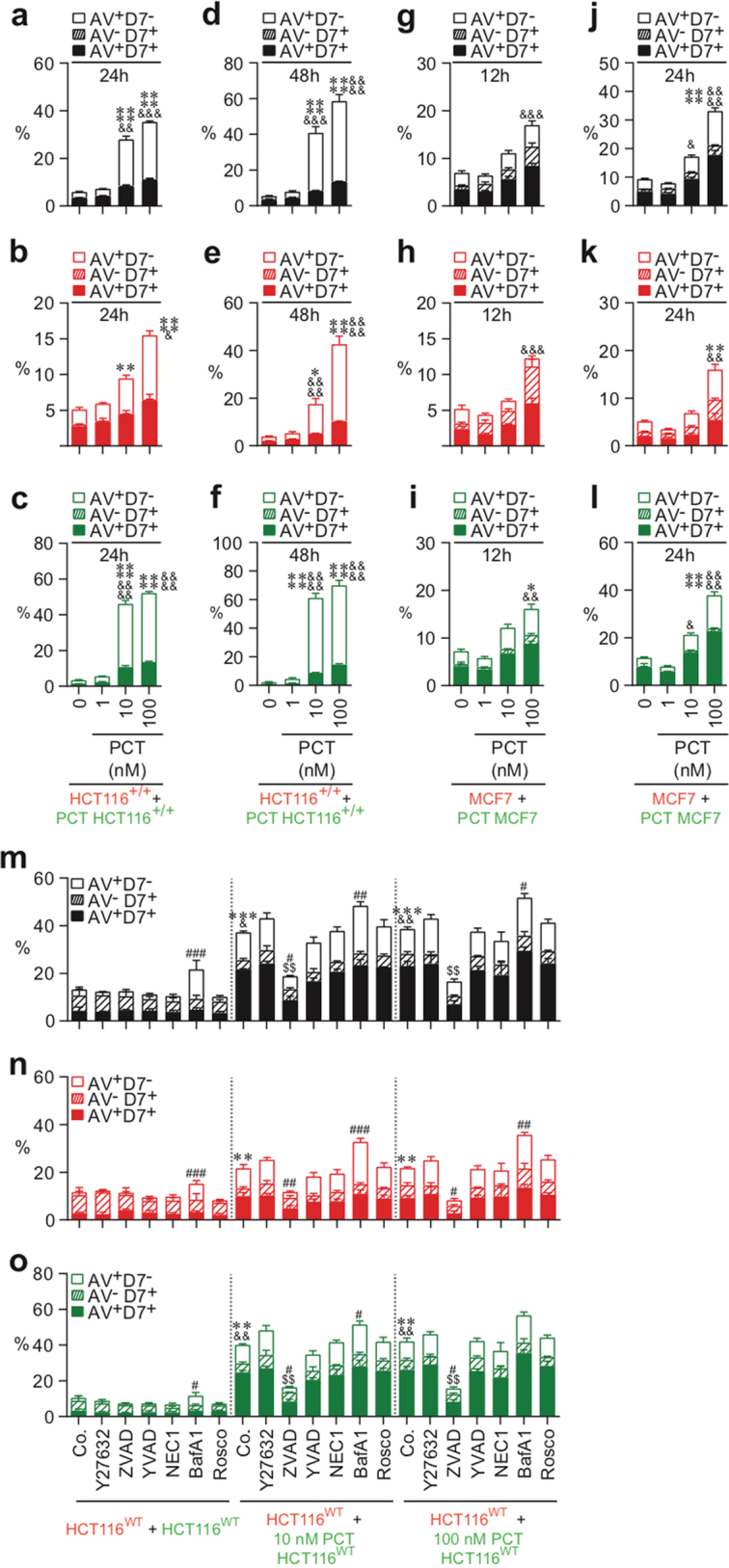
Quantitative imaging flow-cytometric detection of CAD modalities induced by paclitaxel. The cell death profiling was performed after co-cultures of untreated (red) CMTMR-labeled HCT116^WT^, HCT116^+/+^, or MCF7 cells with untreated (green) CMFDA-labeled cells or with paclitaxel (PCT)-treated (green) CMFDA-labeled HCT116^WT^, HCT116^+/+^, or MCF7 cells. Co-cultures have been performed during 12 h (**g**–**i**), 24 h (**a**–**c**, **j**–**o**) or 48 h (**d**–**f**) with the indicated concentrations of PCT in the presence or absence of death effector inhibitors. Then cells were sequentially labeled for the simultaneous detection of type I, II, or III cell death as previously described. Means ± SEM are indicated (*n* = 3). For **a**–**f**, asterisk (*) is used for the comparison of “HCT116^+/+^+PCT-treated HCT116^+/+^” with “HCT116^+/+^+0 nM HCT116^+/+^” for AV^+^D7^−^, ampersand (&) is used for the comparison of “HCT116^+/+^+PCT-treated HCT116^+/+^” with “HCT116^+/+^+0 nM HCT116^+/+^” for D7^+^. For **g**–**l**, asterisk (*) is used for the comparison of “MCF7+PCT-treated MCF7” cells with “MCF7+0 nM MCF7” for AV^+^D7^−^, and ampersand (&) is used for the comparison of “MCF7+PCT-treated MCF7” with “MCF7+0 nM MCF7” for D7^+^. For **m**–**o**, asterisk (*) is used for the comparison of “HCT116^WT^+PCT-treated control (Co.) HCT116^WT^” with “HCT116^WT^+control (Co.) HCT116^WT^” for AV^+^D7^−^, ampersand (&) for the comparison of “HCT116^WT^+PCT-treated control (Co.) HCT116^WT^” cells with “HCT116^WT^+control (Co.) HCT116^WT^” cells for D7^+^, hash (#) for the comparison of inhibitor-treated cells with respective control cells for AV^+^D7^−^, and dollar symbol ($) for the comparison of inhibitor-treated cells with respective control cells for D7^+^. *^, #, &^*p* < 0.05; **^, ##, $$, &&^*p* < 0.01; ***^, ###, &&&^*p* < 0.001; and ****^, &&&&^*p* < 0.0001

**Fig. 4:**
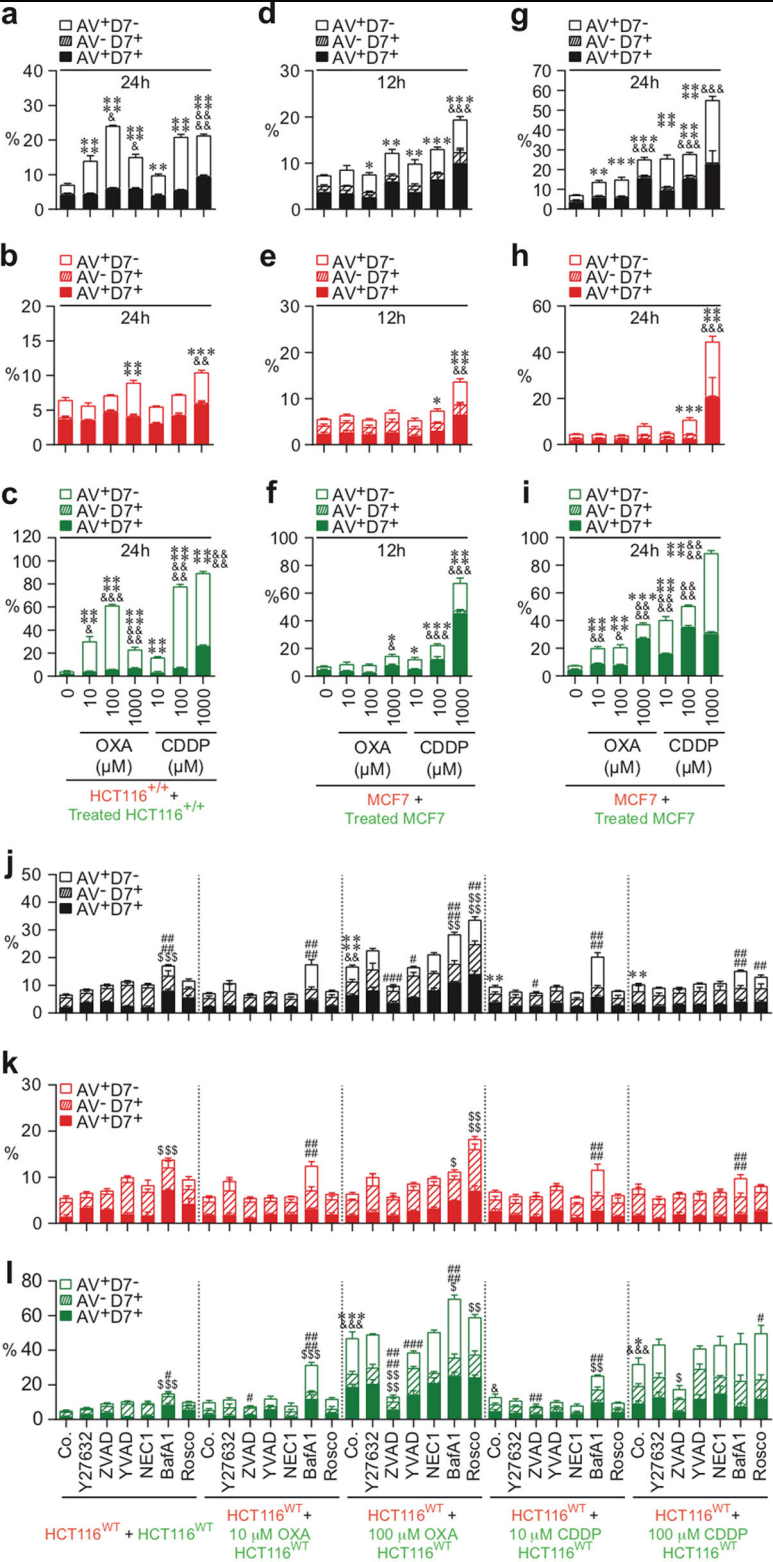
Quantitative imaging flow-cytometric detection of CAD modalities induced by oxaliplatin or cisplatin. The cell death profiling was performed after co-cultures of untreated (red) CMTMR-labeled HCT116^WT^, HCT116^+/+^, or MCF7 cells with untreated (green) CMFDA-labeled cells or with oxaliplatin (OXA)-treated or cisplatin (CDDP)-treated (green) CMFDA-labeled HCT116^WT^, HCT116^+/+^, or MCF7 cells. Co-cultures have been performed during 12 h (**d**–**f**) and 24 h (**a**–**c**, **g**–**l**) with the indicated concentrations of OXA or CDDP in the presence or absence of death effector inhibitors. As previously described, cells were labeled for the simultaneous detection of type I, II, or III cell death. Means ± SEM are indicated (*n* = 3). For **a**–**c**, asterisk (*) is used for the comparison of “HCT116^+/+^+OXA- or CDDP-treated HCT116^+/+^” with “HCT116^+/+^+0 nM HCT116^+/+^” for AV^+^D7^−^, hash (&) is used for the comparison of “HCT116^+/+^+OXA- or CDDP-treated HCT116^+/+^” with “HCT116^+/+^+0 nM HCT116^+/+^” for D7^+^. For **d**–**i**, asterisk (*) is used for the comparison of “MCF7+OXA- or CDDP-treated MCF7” with “MCF7+0 nM MCF7” cells for AV^+^D7^−^ and ampersand (&) is used for the comparison of “MCF7+OXA- or CDDP-treated MCF7” with “MCF7+0 nM MCF7” for D7^+^. For **j**–**l**, asterisk (*) is used for comparison of “HCT116^WT^+OXA- or CDDP-treated control (Co.) HCT116^WT^” with “HCT116^WT^+control (Co.) HCT116^WT^” for AV^+^D7^−^, ampersand (&) for the comparison of “HCT116^WT^+OXA- or CDDP-treated control (Co.) HCT116^WT^” with “HCT116^WT^+control (Co.) HCT116^WT^” for D7^+^, hash (#) for the comparison of inhibitor-treated cells with respective control cells for AV^+^D7^−^, and dollar symbol ($) for the comparison of inhibitor-treated cells with respective control cells for D7^+^. *^, #, $, &^*p* < 0.05; **^, ##, $$, &&^*p* < 0.01; ***^, ###, $$$, &&&^*p* < 0.001; and ****^, ####, $$$$, &&&&^*p* < 0.0001

To further evaluate whether chemotherapeutic agents may also eliminate cancer cells by eliciting distinct cell death modalities, we examined the cell death features triggered by paclitaxel (PCT) (a taxane currently used in breast cancer treatment) and by oxaliplatin (OXA) and cisplatin (CDDP), two platinum salts that are frequently used to treat colorectal and non-small cell lung cancers, respectively. Thus CMFDA-labeled HCT116^+/+^ cells (Fig. 3a–f), HCT116^WT^ (Fig. 3m–o), or MCF7 cells (Fig. 3g–l) were treated with the indicated concentrations of PCT (Fig. 3), OXA, or CDDP (Fig. 4) during 24 h, then mixed at a 1:1 ratio with CMTMR-labeled cells, and cultured for 12, 24, or 48 h in the absence or presence of cell death inhibitors. Multispectral imaging flow-cytometric analysis revealed that, after the treatment with PCT, both untreated CMTMR^+^ cells (Fig. 3b, e, h, k) and PCT-treated CMFDA^+^ cells (Fig. 3c, f, i, l) were eliminated in a dose-dependent manner, by apoptosis (as compared to control cells (Fig. 3a–l)). Accordingly, the pan-caspase inhibitor Z-VAD-fmk inhibited the exposure of PS on the outer plasma membrane leaflet of both untreated CMTMR^+^ cells and PCT-treated CMFDA^+^ cells (Fig. 3m–o). Co-cultures of untreated CMTMR-labeled MCF7 cells with CMFDA-labeled MCF7 cells that had been treated with PCT during 24 h revealed after 12 h (Fig. 3g–i) or 24 h (Fig. 3j–l) that, in the absence of caspase-3, untreated CMTMR^+^ cells and PCT-treated CMFDA^+^ cells were mainly eliminated through necrosis (as revealed by the detection of untreated and treated A^−^D7^+^ or A^+^D7^+^ MCF7 cells (Fig. 3j–l)). In contrast to PCT, which induced marked bystander killing (Fig. 3), OXA and CDDP had a less strong bystander effect (Fig. 4). Thus the fraction of OXA- or CDDP-treated CMFDA^+^ cells that underwent apoptosis was larger than that of untreated CMTMR^+^ cells (Fig. 4a–c, j–l). These processes, which were inhibited by the pan-caspase inhibitor Z-VAD-fmk, indicate that a fraction of single HCT116^WT^ cells underwent apoptosis after treatment with OXA or CDDP (Fig. 4j–l). In the absence of caspase-3 (in MFC7 cells), these treatments also killed cancer cells to a lower extent, mainly through a necrotic process (Fig. 4d–i). We observed that PCT-, OXA-, and CDDP-treated HCT116^WT^ and HCT116^+/+^ cells and neighboring cells of PCT-treated cells accumulated in the G2/M phase of the cell cycle (Supplementary Figures 3 and 4). Moreover, OXA- and CDDP-treated MCF7 cells accumulated in the S phase (Supplementary Figure 4). In addition, pharmacological inhibition of caspases (with Z-VAD-fmk) and of Cdk1 (with Rosco) failed to interfere with this G2/M arrest (Supplementary Figures 3m-3o and 4m-4o), indicating that killing of both treated and neighboring cells was not associated with cell cycle progression. These results demonstrate that chemotherapeutic agents such as PCT can exert their cytotoxic effects on cancer cells through direct and indirect cell-autonomous lethal pathways.

### IR, PCT, OXA, and CDDP also induce NCAD

**Fig. 5:**
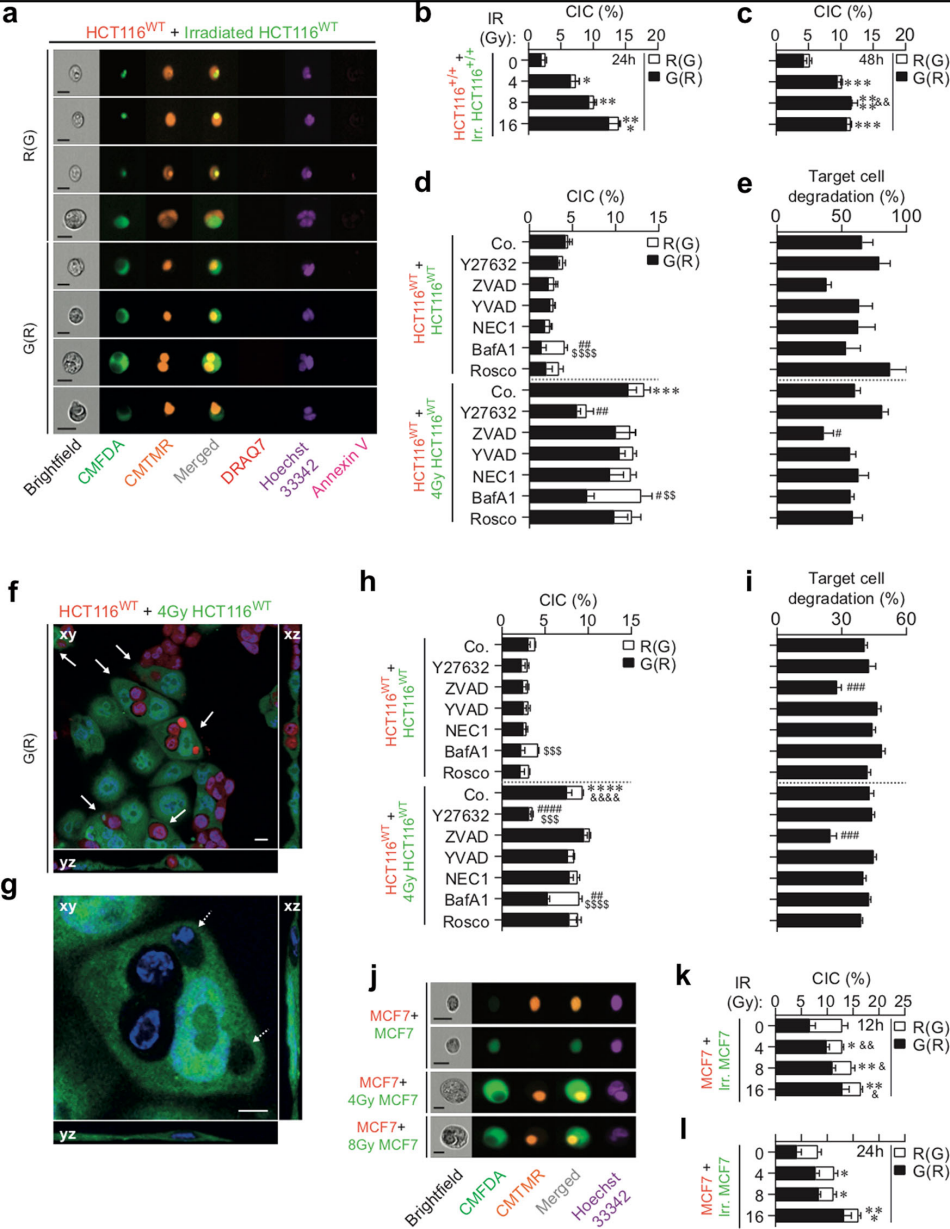
Detection of γ-irradiation–elicited NCAD modalities by quantitative imaging flow cytometry and confocal fluorescence microscopy. **a**–**l** Simultaneously to the detection of CAD mechanisms in Fig. 2, cell-in-cell structures and target cell degradation were determined by quantitative imaging (**a**–**e** and **j**–**l**) and confocal fluorescent microscopy (**f**–**i**) after 24-h (**a**, **b**, **d**–**i**, **l**), 48-h (**c**), or 12-h (**j**, **k**) co-culture of untreated (red) CMTMR-labeled HCT116^WT^ cells (**a**, **d**–**i**), HCT116^+/+^ cells (**b**, **c**), or MCF7 cells (**j**–**l**) with, respectively, (green) CMFDA-labeled HCT116^WT^ cells (**a**, **d–i**), HCT116^+/+^ cells (**b**, **c**), or MCF7 cells (**j**–**l**) that have been irradiated or not with 4 Gy of γ-ionizing radiation. Then (red) CMTMR-labeled HCT116 cells internalizing (green) CMFDA-labeled HCT116 cells (noted R(G)), and (green) CMFDA-labeled HCT116 cells internalizing (red) CMTMR-labeled HCT116 cells (noted G(R)) were detected and quantified. Representative images of quantitative imaging are shown in (**a**, **j**) (scale, 20 μm). Representative images (**a**, **f**, **g**, **j**) and frequencies of cell-in-cell (CIC) structures (**b**–**d**, **h**, **k**, **l**) and target cell degradation (**e**, **i**) were obtained and quantified by quantitative imaging flow cytometry (**a**–**e** and **j**–**l**) and confocal microscopy (**f**–**i**). White arrows indicate CIC structures and white dotted arrows target cell degradation as observed by confocal microscopy (**f**, **g**) (scale bar = 10 μm). Frequencies of CIC structures showing R(G), and G(R) (**h**) and target cell degradation (**i**) have been determined (means ± SEM, *n* = 3). For **b**, **c**, asterisk (*) is used for the comparison of “HCT116^+/+^+Irr.HCT116^+/+^” with “HCT116^+/+^+0 Gy HCT116^+/+^” for G(R), and ampersand (&) is used for the comparison of “HCT116^+/+^+Irr. HCT116^+/+^” with “HCT116^+/+^+0 Gy HCT116^+/+^” cells for R(G). For **d**, **h**, asterisk (*) is used for the comparison of “HCT116^WT^+4 Gy control (Co.) HCT116^WT^” with “HCT116^WT^+control (Co.) HCT116^WT^” for G(R), ampersand (&) for comparison of “HCT116^WT^+4 Gy control (Co.) HCT116^WT^” cells with “HCT116^WT^+control (Co.) HCT116^WT^” for R(G), hash (#) for the comparison of inhibitor-treated cells with control cells for G(R), and dollar symbol ($) for the comparison of inhibitor-treated cells with respective control cells for R(G). For **e**, **i**, hash (#) is used for the comparison of inhibitor-treated cells with respective control cells for target cell degradation. For **k**, **l**, asterisk (*) is used for the comparison of “MCF7+Irr. MCF7” with “MCF7+0 Gy MCF7” for G(R) and ampersand (&) is used for the comparison of “MCF7+Irr. MCF7” with “MCF7+0 Gy MCF7” for R(G). *^, #, &^*p* < 0.05; **^, ##, &&^*p* < 0.01; ***^, ###, $$$^*p* < 0.001; and ****^, ####, $$$$, &&&&^*p* < 0.0001

**Fig. 6:**
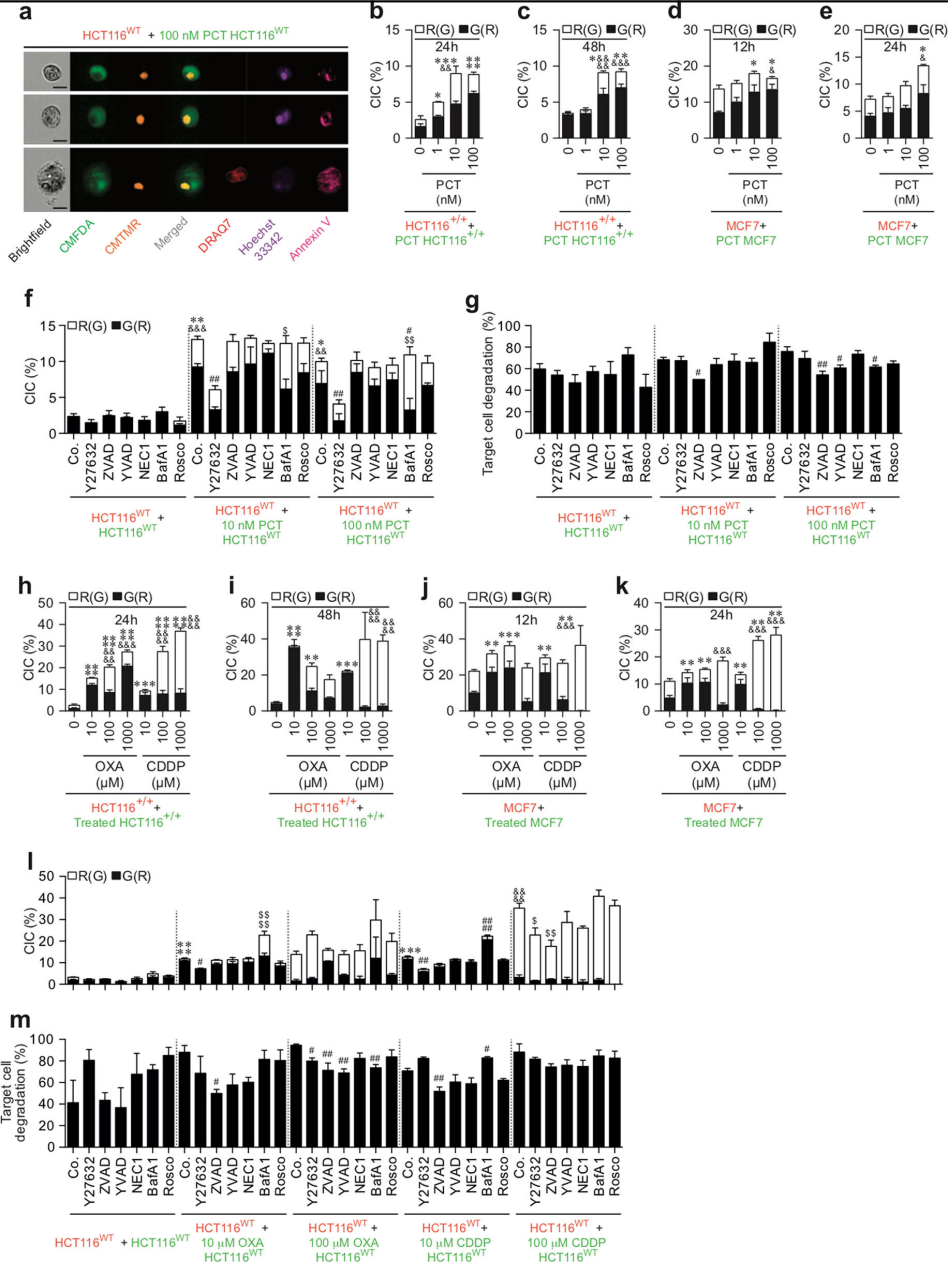
Quantitative imaging flow-cytometric detection of NCAD modalities triggered by paclitaxel, oxaliplatin, or cisplatin. Simultaneously to the detection of CAD in Figs. 3 and 4, NCAD mechanisms were determined with the indicated concentrations of PCT, OXA, and CDDP and in the presence or absence of the indicated inhibitors after co-cultures of untreated (red) CMTMR-labeled cells (HCT116^WT^ cells (**a**, **f**, **g**, **l, m**), HCT116^+/+^ cells (**b**, **c**), or MCF7 cells (**d**, **e**)) with untreated (green) CMFDA-labeled isogenic cells and after co-cultures of untreated (red) CMTMR-labeled cells (HCT116^WT^ cells (**a**, **f**, **g**, **l**, **m**), HCT116^+/+^ cells (**b**, **c**), or MCF7 cells (**d**, **e**)) with PCT-, OXA-, or CDDP-treated (green) CMFDA-labeled isogenic cells. The detection of cell-in-cell structures (**a**–**f**, **h**–**l**) and target cell degradation (**g**, **m**) were performed as indicated in the legend of Fig. 5. Frequencies are shown (means ± SEM, *n* = 3). For **b**, **c**, asterisk (*) is used for the comparison of “HCT116^+/+^+PCT-treated HCT116^+/+^” with “HCT116^+/+^+0 nM HCT116^+/+^” for G(R), ampersand (&) is used for the comparison of “HCT116^+/+^+PCT-treated HCT116^+/+^” with “HCT116^+/+^+0 nM HCT116^+/+^” for R(G). For **d**, **e**, asterisk (*) is used for comparison of “MCF7+PCT-treated MCF7” with “MCF7+0 nM MCF7” cells for G(R), ampersand (&) is used for the comparison of “MCF7+PCT-treated MCF7” with “MCF7+0 nM MCF7” cells for R(G). For **f**, asterisk (*) is used for the comparison of “HCT116^WT^+PCT-treated HCT116^WT^” with “HCT116^WT^+control (Co.) HCT116^WT^” for G(R), ampersand (&) for the comparison of “HCT116^WT^+PCT-treated HCT116^WT^” with “HCT116^WT^+control (Co.) HCT116^WT^” for R(G), hash (#) for the comparison of inhibitor-treated cells with control cells for G(R), and dollar symbol ($) for the comparison of inhibitor-treated cells with respective control cells for R(G). For **g**, **m**, hash (#) is for the comparison of inhibitor-treated cells with respective control cells for target cell degradation. For **h**, **i**, asterisk (*) is used for the comparison of “HCT116^+/+^+OXA- or CDDP-treated HCT116^+/+^” with “HCT116^+/+^+0 nM HCT116^+/+^” cells for G(R), and ampersand (&) is used for the comparison of “HCT116^+/+^+OXA- or CDDP-treated HCT116^+/+^” with “HCT116^+/+^+0 nM HCT116^+/+^” for R(G). For **j**, **k**, asterisk (*) is used for the comparison of “MCF7+OXA- or CDDP-treated MCF7” with “MCF7+0 nM MCF7” for G(R) and ampersand (&) is used for the comparison of “MCF7+OXA- or CDDP-treated MCF7” with “MCF7+0 nM MCF7” for R(G). For **l**, asterisk (*) for the comparison of “HCT116^WT^+OXA- or CDDP-treated HCT116^WT^” cells with “HCT116 ^WT^+control (Co.) HCT116 ^WT^” cells for G(R), ampersand (&) for the comparison of “HCT116 ^WT^+OXA- or CDDP-treated HCT116^WT^” with “HCT116 ^WT^+control (Co.) HCT116^WT^” cells for R(G), hash (#) for the comparison of inhibitor-treated cells with control cells for G(R) and dollar symbol ($) for comparison of inhibitor-treated cells with respective control cells for R(G). *^, #, $, &^*p* < 0.05; **^, ##, $$, &&^*p* < 0.01; *** or ^&&&^*p* < 0.001; and ****^, $$$$, &&&&^*p* < 0.0001

**Fig. 7:**
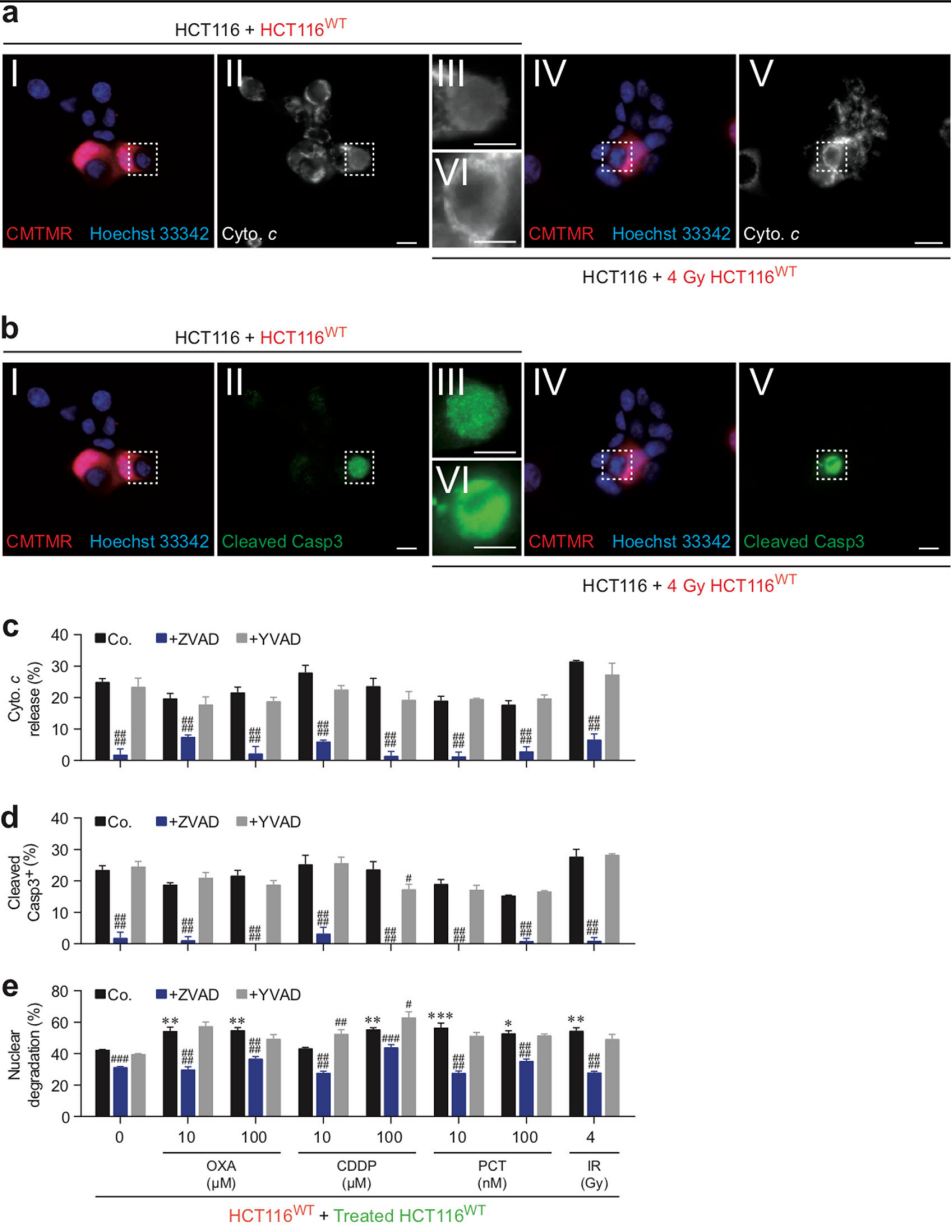
Detection of biochemical features of engulfed cells detected after OXA, CDDP-PCT or IR treatment. **a**, **b** Representative fluorescent images of cytochrome *c* release, cleavage of CASP3, and nuclear degradation in engulfed HCT116^WT^ cells detected after 24-h co-culture of untreated HCT116^WT^ cells with untreated (red) CMTMR-labeled HCT116^WT^ cells (**a**I–**a**III, **b**I–**b**III) and after 24-h co-culture of untreated HCT116^WT^ cells with (red) CMTMR-labeled HCT116^WT^ cells that have been irradiated with 4 Gy of γ-ionizing radiation in the presence or absence of 100 μM Z-VAD-fmk (ZVAD) or 100 μM Y-VAD-cmk (YVAD) (**a**IV–**a**VI, **b**IV–**b**VI) are shown. Frequencies of cells showing cytochrome *c* release (**c**), cleaved CASP3 (**d**), and nuclear degradation (**e**) are shown (means ± SEM, *n* = 3). For **c**–**e**, asterisk (*) is used for “HCT116^WT^+OXA-treated HCT116^WT^”, “HCT116^WT^+CDDP-treated HCT116^WT^”, “HCT116^WT^+PCT-treated HCT116^WT^”, and “HCT116^WT^+Irradiated (IR) HCT116^WT^” with “HCT116^WT^+control (Co.) HCT116^WT^” for nuclear degradation and hash (#) is used for the comparison of inhibitor-treated cells with respective control cells for cytochrome *c* release, cleaved caspase-3, or nuclear degradation. *^, #^*p* < 0.05; **^, ##^*p* < 0.01; ***^, ###^*p* < 0.001; and ^####^*p* < 0.0001

In parallel, we examined in the same co-cultures the ability of irradiated or PCT-, OXA-, or CDDP-treated CMFDA^+^ cells to engulf or invade neighboring cells, two cellular processes required for induction of cellular cannibalism-associated cell death (e.g., as cellular cannibalism or phagoptosis) or cell-in-cell invasion-elicited cell death (e.g., as entosis or emperitosis). Multispectral imaging flow-cytometric analysis revealed that irradiated or treated CMFDA^+^ HCT116^WT^, HCT116^+/+^, or MCF7 cells triggered the engulfment of neighboring cells after 12, 24, and 48 h of co-culture (as revealed by the internalization of “target” CMTMR^+^ (red) cells by IR-, PCT-, OXA-, or CDDP-treated CMFDA^+^ (green) cells detected with multispectral imaging flow cytometry (Figs. 5a–e, j–l and 6) and confocal microscopy (Fig. 5f–h)). This process was repressed by the inhibitor of ROCK1 (Y27632) but was not affected by the pan-caspase inhibitor Z-VAD-fmk (ZVAD) (Fig. 5d, h and 6f, l), indicating that the detected cell-in-cell internalization is distinct from phagocytic uptake of apoptotic cells and requires ROCK1 activity. Importantly, the cell death profiling analysis allowed us to distinguish between live cell engulfment and phagocytosis of apoptotic CMFDA^+^ cells by live CMTMR^+^ cells that follows apoptosis induced by treatment with BafA1 (Figs. 5d and 6f) or with high concentrations (100 μM and 1 mM) of OXA or CDDP (Fig. 6h–l). We then evaluated the fate of engulfed CMTMR^+^ HCT116^WT^ cells and those of irradiated or PCT-, OXA-, or CDDP-treated engulfing CMFDA^+^ HCT116^WT^ cells. We observed that approximately 50% of engulfed CMTMR^+^ HCT116^WT^ cells exhibited signs of cellular degradation, as revealed by a decreased size of internalized cells detected with multispectral imaging flow cytometry (Figs. 5a, e and 6a, g, m) and the DNA content loss of internalized cells detected by confocal microscopy (Fig. 5f, g, i). This process was partially reduced in the presence of the pan-caspase inhibitor Z-VAD-fmk, demonstrating that the death of engulfed cells occurs through caspase-dependent and caspase-independent mechanisms (Figs. 5i and 6g). To explore the lethal mechanisms through which engulfed cells are eliminated, we detected and quantified by fluorescent microscopy the release of cytochrome *c*, caspase-3 cleavage, and nuclear fragmentation in engulfed cells obtained after homotypic cultures of HCT116^WT^ cells. Within cannibal cells elicited by IR, PCT, OXA, or CDDP treatment, the engulfed cells exhibited signs of apoptosis such as cytochrome *c* release, activating cleavage of caspase-3 and nuclear degradation (Fig. 7a–e). We also determined the impact of the pan-caspase inhibitor Z-VAD-fmk and the caspase-1 inhibitor YVAD-cmk on these apoptotic features. Fluorescence microscopy revealed that Z-VAD-fmk (but not YVAD-cmk) impaired release of cytochrome *c* and activation of caspase-3 in engulfed cells (Fig. 7c, d) but only partially inhibited nuclear degradation with such cells (Fig. 7e). These results confirm those obtained using multispectral flow imaging and clearly demonstrate that the death of engulfed cells requires the activation of both caspase-dependent and -independent mechanisms. In addition, the vast majority of cannibal cells did not expose PS and did not exhibit loss of the integrity of their plasma membrane after anticancer treatments (Supplementary Figures 5a-5d) underscoring the fact that, after IR-, PCT-, OXA-, or CDDP-mediated cell engulfment, the internalized cells succumb to death without compromising the viability of the engulfing cells. Altogether, these results show that IR and major chemotherapeutic agents (such as PCT, OXA, and CDDP) can simultaneously induce CAD and NCAD in both treated and untreated cells.

### The transcriptional activity of p53 tumor-suppressor protein distinctly impacts the execution of CAD and NCAD elicited by anticancer treatment

**Fig. 8:**
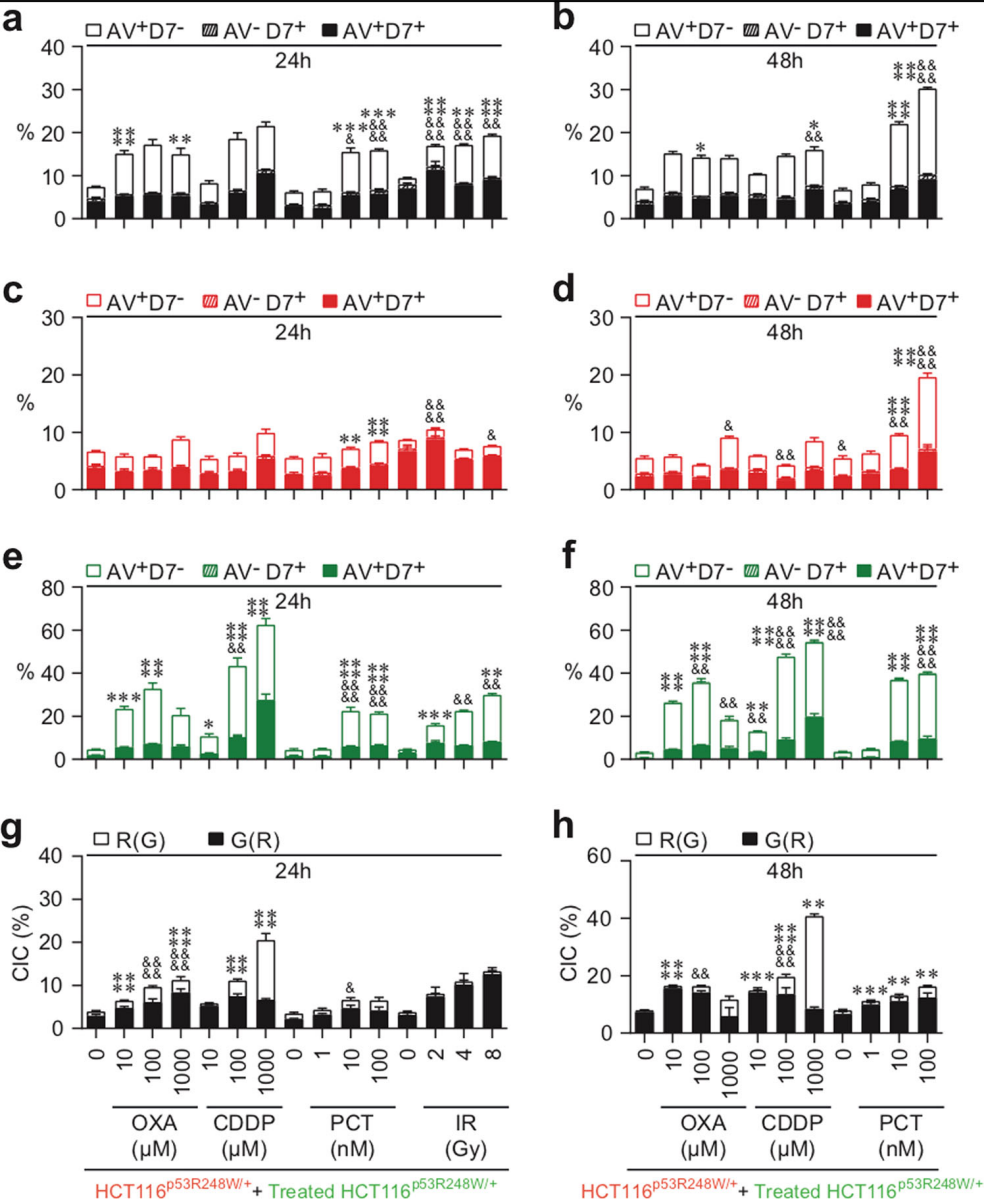
CAD and NCAD modalities elicited by chemotherapies and radiotherapy are distinctly regulated by p53 transcriptional activity. Quantitative imaging flow-cytometric detection of CAD and NCAD modalities was performed after 24 h (**a**, **c**, **e**, **g**) and 48 h (**b**, **d**, **f**, **h**) co-cultures of untreated (red) CMTMR-labeled HCT116 ^p53R248W/+^ cells with untreated (green) CMFDA-labeled HCT116 ^p53R248W/+^ cells, or with OXA-, CDDP-, PCT- or IR-treated (green) CMFDA-labeled HCT116 ^p53R248W/+^ cells. Concentrations of OXA, CDDP, or PCT or doses of γ-irradiation used are indicated. The frequencies of AV^+^D7^−^ cells, AV^−^D7^+^ cells, and AV^+^D7^+^ cells were determined as previously described on total cell population (as revealed by CMTMR^+^ or CMFDA^+^ HCT116 ^p53R248W/+^ cells (**a**, **b**)), on untreated (red) CMTMR^+^ HCT116 ^p53R248W/+^ cells (**c**, **d**), on untreated (green) CMFDA^+^ HCT116 cells (**e**, **f**), and on treated (green) CMFDA^+^ HCT116 ^p53R248W/+^ (**e**, **f**). Means ± SEM are indicated (*n* = 3). For **a**–**f**, asterisk (*) is used for the comparison of “HCT116 ^p53R248W/+^+OXA- or CDDP- or PCT- or IR-treated HCT116 ^p53R248W/+^” with “HCT116^+/+^+OXA- or CDDP- or PCT- or IR-treated HCT116^+/+^” (from Figs. 2c–e, 3a–f, and 4a-c) for AV^+^D7^−^, and ampersand (&) is used for the comparison of “HCT116^R248W/+^+OXA- or CDDP- or PCT- or IR-treated HCT116 ^p53R248W/+^” with “HCT116^+/+^+OXA- or CDDP- or PCT- or IR-treated HCT116^+/+^” (from Figs. 2c–e, 3a–f, and 4a–c) for D7^+^. For Fig. 7g, h, asterisk (*) is used for the comparison of “HCT116 ^p53R248W/+^+OXA- or CDDP- or PCT- or IR-treated HCT116 ^p53R248W/+^” with “HCT116^+/+^+OXA-or CDDP- or PCT- or IR-treated HCT116^+/+^” (from Fig. 5b, c) for G(R), and ampersand (&) is used for comparison of “HCT116 ^p53R248W/+^+OXA- or CDDP- or PCT- or IR-treated HCT116 ^p53R248W/+^” with “HCT116^+/+^+OXA- or CDDP- or PCT- or IR-treated HCT116^+/+^” (from Fig. 5b, c) for R(G). *^, &^*p* < 0.05; **^, &&^*p* < 0.01; ***p < 0.001; and ****^, &&&&^*p* < 0.0001

Considering that p53 inactivation is a major cellular factor involved in the resistance of tumor cells to anticancer treatments^26^, we analyzed the impact of inhibition of p53 transcriptional activity on the induction of CAD and NCAD. Thus we examined the influence of a dominant-negative mutation at codon 248 in one allele of p53 (p53R248W) on the execution of CAD and NCAD elicited by IR, PCT, CDDP, or OXA treatment. In parallel to previous experiments (Figs. 2c–h, 3a–f, 4a–c, 5b, c, and 6b, c, h, i), CMFDA-labeled HCT116 ^p53R248W/+^ cells were irradiated with different doses (Fig. 8a, c, e, g) or treated with the indicated concentrations of PCT, OXA, or CDDP (Fig. 8a–h) for 24 h, then mixed at a 1:1 ratio with CMTMR-labeled cells, and co-cultured for 24 h (Fig. 8a, c, e, g) or 48 h (Fig. 8b, d, f, h). As described above, PS exposure, plasma membrane integrity, and DNA content of each cellular partner were then analyzed using multispectral imaging flow cytometry. As expected, we observed a significant inhibition of PCT-, CDDP-, and OXA-elicited apoptosis of irradiated or treated HCT116 ^p53R248W/+^ cells (Fig. 8a, e). In addition, p53 inactivation impaired PCT-induced apoptosis of untreated HCT116 ^p53R248W/+^cells (Fig. 8c), as compared to control irradiated or treated HCT116 ^+/+^cells (Figs. 2c–h, 3a–f, 4a–c, 5b, c, and 6b, c, h, i). Although, p53 inactivation had no impact on IR-, PCT-, and CDDP-mediated cellular cannibalism, OXA-induced cellular cannibalism was repressed when transcriptional activity of p53 was inhibited (Fig. 8g, h), as compared to control irradiated or treated HCT116^+/+^ cells (Fig. 5d and 6b, c, h, i). p53 transcriptional inactivation similarly impacted the progression through the cell cycles of IR-, PCT-, OXA-, and CDDP-treated HCT116 ^p53R248W/+^cells (Supplementary Figure 6), as compared to control cells (Figs. 2c–h, 3a–f, and 4a–c). Altogether, these results demonstrated that the transcription factor p53 distinctly modulates the induction of NCAD and emphasizes the conclusion that the stimulation of cannibalistic activity of p53-mutated cancer cells by anticancer chemotherapies (such as PCT and CDDP) or radiotherapy should be considered as a treatment option.

## Discussion

Using a novel quantitative multispectral imaging flow-cytometry-based platform and co-culture experiments, we could provide evidence suggesting that IR and chemotherapeutic agents (such as PCT, OXA, and CDDP) can simultaneously trigger CAD and NCAD in both treated and untreated cancer cell populations. Thus, for the first time, we observed that, after IR, a fraction of irradiated cancer cells is eliminated through CAD, while another fraction of the irradiated cancer cell population engulfed and killed neighboring cells via both caspase-dependent and -independent death mechanisms. Similarly, OXA and CDDP triggered killing of some treated cancer cells through cell-autonomous apoptosis and cellular cannibalism. Interestingly, PCT not only simultaneously induced CAD and NCAD in distinct fractions of the treated cell population but also caused the apoptosis of untreated cells co-cultured with PCT-treated cells. Consistent with prior reports^18, 27^, these results reveal the individual variability of cell responses to death-inducing stimuli and the coexistence of many types of cell death subroutines in the same cell population. Thus the global effect of cell death inducers appears to be more related to the sum of multiple lethal processes than the consequence of the execution of a single-cell death modality. These processes can not only occur through cell-autonomous or non-cell-autonomous mechanisms and act directly on treated cells but also through bystander effect on untreated neighboring cells. Altogether, these results demonstrate the existence of the heterogeneity of cell death responses to anticancer treatments and may thus help to explain the absence of a link between the induction of CAD (such as apoptosis) and the clonogenic survival of cancer cells observed after IR^18^. Considering that IR, PCT, and OXA are well-known immunogenic cell death inducers^28^, the relative contribution of each CAD and NCAD to release danger-associated molecular patterns or other immunostimulatory factors and to favor the development of tumor-specific immune responses remains to be determined.

Intriguingly, we observed that the transcriptional activity of tumor-suppressor p53 distinctly affects the execution of both CAD and NCAD. The inactivation of p53 transcriptional activity impaired OXA-induced cellular cannibalism but failed to alter IR-, PCT-, and CDDP-mediated cellular cannibalism. These results support the interpretation that NCAD can be induced in cancer cells harboring or not dominant-negative mutants of p53 and should render these cells more sensitive to anticancer treatment. These results underscore the importance for further systematic evaluation of the prevalence of CAD and NCAD both in physiological or pathological situations. Phagoptosis has been detected during the development of the nematode *Caenorhabditis elegans*^29, 30^. In mammals, phagoptosis has not only been involved in cellular and tissue homeostasis (e.g., during brain development)^12^ but also during the inflammation and neurodegeneration^13^. Recently, the gene *Ced-10* has been shown to contribute to the killing of primordial germ cells by controlling the cannibalistic activity of endodermal cells^31^. Considering the normal embryonic development and near-normal survival of mice lacking apoptotic pathway components (such as *Apaf*^*−/−*^, *Bax*^*−/−*^, *Bak*^*−/−*^, or double knockout mice)^32, 33^, the relative contribution of NCAD to embryonic development, cellular homeostasis, and cell loss remains to be explored and the relationship between the induction of CAD and NCAD in vivo needs to be investigated. In addition, cell death profiling analysis should facilitate the characterization of alternative cell death mechanisms (such as keratinocyte death by cornification^34^ or entosis^7^). More importantly, the mechanistic elucidation of NCAD should help to revisit several concepts and signaling models that have been proposed to explain cell death processes. Particular attention should be paid to partial biological effects or a shift from one cell death modality to another cell death that have been detected when cell death effectors (such as caspases) are inhibited^35–38^. The relative contribution of CAD and NCAD to the shift from apoptosis to necroptosis described when Fas-overexpressing L929 fibrosarcoma cells were stimulated by agonistic anti-Fas antibody in the presence of the pan-caspase inhibitor Z-VAD-fmk could be re-analyzed for evaluation of the cell death profile using quantitative multispectral imaging flow cytometry. Our results also suggest that the quantitative and qualitative variations of cell death profiles elicited by different types of anticancer treatment should differentially impact biological and immunological tumor responses to anticancer treatment. Further molecular characterization defining the impact of each cell death modality on the global cancer cell fate in vitro and on tumor growth in vivo should help to unravel the exact contribution of CAD and NCAD to anticancer treatment.

Taken together, our results demonstrate the coexistence of CAD (such as type I, II, and III cell death modalities) with NCAD (as the proposed type IV cell death modality)^6^. Future studies aiming at the pharmacological or genetic manipulation of cell death effectors (such as proapoptotic BCL-2 family members, BAX and BAK) should contribute to mitigate the notion of “the point of no return,” which has been proposed as the central cellular event for cell fate decision, considering lethal signaling pathways as a linear cascade of molecular events coming into play in a cell-autonomous fashion^5^. Indeed, future analyses must contemplate the heterogeneity of cellular responses to anticancer agents and IR and admit the possibility that non-cell autonomous and bystander effects might play a major role in determining the efficacy of cancer treatments.

## Material and methods

### Chemicals, cell lines, and culture conditions

Unless otherwise indicated, chemicals and acetyl-Tyr-Val-Ala-Asp-chloromethylketone (Ac-YVAD-cmk) were purchased from Sigma-Aldrich. Antibiotics, media, and supplements for cell culture were obtained from Life Technologies. Z-VAD-fmk was from Bachem and recombinant mouse tumor necrosis factor-alpha from R&D systems. Human colon carcinoma WT HCT116 (HCT116^WT^) cells, isogenic dominant-negative p53R248W-expressing HCT116 (HCT116^p53R258W/+^) cells, WT p53 control HCT116 (HCT116^+/+^) cells, human MCF7 breast cancer cells, and murine fibrosarcoma cell line L929 were cultured in Dulbecco’s modified Eagle’s medium. HCT116^p53R258W/+^ cells and HCT116^+/+^ cells are isogenic pairs of cells obtained from WT HCT116 (HCT116^WT^). Jurkat T cells were maintained in RPMI medium. All the media were supplemented with 10% heat-inactivated fetal bovine serum (FBS), 10 mM HEPES buffers, 2 mM L-glutamine, 10 U/mL penicillin sodium, and 10 μg/mL streptomycin sulfate.

### Irradiation and treatment with chemotherapeutic agents

Cells were seeded in 6-well plates, 12-well plates, or 25 cm^2^ flasks for 24 h. Then adherent cells were treated with different concentrations of PCT, OXA, or CDDP or irradiated at the indicated dose with gamma-ray irradiator IBL-637 (Cs^137^, 1 Gy/min, gamma CIS-BioInternational, IBA, Saclay, France) during 24 h.

### CellTracker™ fluorescent probes labeling and co-culture experiments

Upon the removal of the culture medium, cancer cells were incubated with prewarmed medium containing 10 μM of CMFDA (green fluorescence) or CMTMR (red fluorescence) (Molecular Probes-Life Technologies) for 45 min at 37 °C. Thereafter, cancer cells were rinsed twice with prewarmed medium and incubated for 45 min at 37 °C. Stained cells were then detached with Tryspin-EDTA solution (Life Technologies), suspended in complete medium, and cultured for the indicated times for cell death profiling or confocal microscopic analysis. Untreated cancer cells were labeled with CMFDA (green fluorescence, CMFDA^+^) or CMTMR (red fluorescence, CMTMR^+^) and treated cancer cells with CMFDA (green fluorescence, CMFDA^+^). The following cell mixtures (at a 1/1 ratio) were performed: untreated CMTMR^+^ cancer cells were mixed with untreated CMFDA^+^ cancer cells or untreated CMTMR^+^ cancer cells were mixed with treated CMFDA^+^ cancer cells. Then cells were co-cultured during the indicated times in the presence or absence of the pharmacological inhibitor of ROCK, Y27632 (30 μM), the pan-caspase inhibitor, Z-VAD-fmk (ZVAD, 100 μM), the inhibitor of caspase-1, Ac-YVAD-cmk (YVAD, 100 μM), the necroptosis inhibitor, NEC1 (30 μM), the inhibitor of the vacuolar type H(+)-ATPase (V-ATPase) inhibiting autophagy, BafA1 (50 nM), and the inhibitor of Cdks with an anti-mitotic activity, Rosco (10 μM).

### Cell death profiling by quantitative flow imaging

After the indicated times of co-cultures, both detached and adherent cells were collected and stained with Hoechst 33342 (10 μg/mL) during 1 h at 37 °C in warmed complete medium. To detect PS exposure and plasma membrane permeability, labeled HCT116 cells were successively incubated with Biotin-AnnexinV (BD Pharmingen) as recommended by the manufacturer, 0.5 μg BV786-Streptavidin (BD Biosciences), and 3 μM DRAQ7 (BioStatus) during 15 min at 25 °C. After washing with phosphate-buffered saline (PBS) solution, samples were immediately analyzed using an imaging flow cytometer FlowSight® (Amnis®, part of EMD Millipore). Data were acquired at a 20× magnification, using the INSPIRE software. The 405, 488, and 561 nm lasers were used for excitation. Brightfield, Annexin V-BV786, DRAQ7, CMFDA, CMTMR, and Hoechst 33342 stainings were detected using, respectively, channels for 420–480, 745–800, 642–745, 480–560, 595–642, and 430–505 nm. At least 1000 events of cells per sample were analyzed. Additional single-labeled controls were prepared to normalize fluorescent signal across different channels. Acquired data were analyzed using the IDEAS analysis software (v6.1; Merck-Millipore). Gating strategy was the following. Cells were gated for focused cells using the Gradient RMS feature. Cells were gated for single cells using the aspect ratio and area features. For the cannibalism detection, cells were gated in the double positive CMFDA^+^ and CMTMR^+^ staining. The imaging flow cytometer FlowSight® provides the images of each events and thus allows to remove from the analysis the artifacts (such as cellular fragments or debris). We determined the sample size required for analyzing the main types of cell death elicited by anticancer treatments (such as apoptosis, necrosis, and cellular cannibal) by calculating the statistical power of our method (statistical power = 0,8; *α* = 0,05; sampling ratio = 1). We found that the number of cellular events that we need to acquire for obtaining statistically significant results is ranging between 300 and 900 cellular events. Accordingly, 1000 cellular events per condition were analyzed in Figs. 2a, b, o–q, 3m–o, 4j–l, 5d, e, and 6f, g, l, m and Supplementary Figures 1e, 2o-2q, 3m-3o, 4j-4l, and 5a-5d. To confirm these results, we then analyzed 10,000 cellular events per condition and validated previous results obtained with imaging flow cytometer as shown in Figs. 2c–n,3a–l, 4a–i, 5b, c, k, l, 6b–e, h–k, and 8a–h. These results were also confirmed using conventional methods such as classical flow cytometry and microscopy (as shown in Fig. 3d–i).

### Flow cytometry and confocal fluorescent microscopy

To detect PS exposure, plasma membrane permeability, and cell cycle progression, cells were after co-culture sequentially labeled with specific fluorescent probes (such as fluorescein isothiocyanate (FITC)-conjugated AnnexinV, propidium iodide (PI), and Hoechst 33342) and analyzed by flow cytometry. Both detached and adherent cells were collected and stained with Hoechst 33345 (10 ug/ml) during 1 h at 37 °C in warmed complete medium. After washing with PBS, HCT116 cells were suspended in 1× binding buffer supplemented with FITC-conjugated Annexin V (BD Biosciences) and PI (1 μg/mL) (Sigma), as per the manufacturer’s instructions. Samples were then analyzed using LSRII flow cytometer (Becton Dickinson) and the FlowJo software v10. For confocal fluorescence microscopy, CMFDA- and CMTMR-labeled HCT116 cells were fixed after co-culture in 3.7% paraformaldehyde–PBS for 15 min. Cells were either counterstained with Hoechst 33342 (Invitrogen) and analyzed for the detection of cell-in-cell structures or permeabilized in 0.1% sodium dodecyl sulfate in PBS and incubated with FBS for 20 min, as previously described^39^. Primary antibodies for detection of cytochrome *c* (#6H2.B4, Thermo Fisher Scientific) or cleaved caspase-3 (Asp175) (#9661, Cell Signaling Technology) were used in PBS containing 1 mg/ml bovine serum albumin and revealed with goat anti-rabbit immunoglobulin G (IgG) conjugated to Alexa 488 fluorochromes or with rabbit anti-mouse IgG conjugated to Alexa 488 fluorochromes from Invitrogen. Then cells were counterstained with Hoechst 33342 (Invitrogen) and analyzed by confocal SPE microscope equipped with Apochromat 63× 1.3 NA and 63× 1.15 NA oil immersion objectives. The Leica Application Suite version 2.6 software was used (Leica Microsystems).

### Western blots

Total cellular proteins were extracted in lysis buffer (containing 1% NP40, 20 mmol/L HEPES, 10 mmol/L KCl, 1 mmol/L EDTA, 10% glycerol, protease, and phosphatase inhibitor tablets). Protein extracts (30 µg) were run on 4–12% NuPAGE® Novex® Bis-Tris gels (Life Technologies) and transferred at 4 °C onto Immobilon polyvinyldifluoride membranes (Thermo Scientific). After blocking, membranes were incubated at 4 °C overnight with primary antibodies specific for: caspase-3 (#9662), cleaved caspase-3 (Asp175) (#9661), Myosin Light Chain 2 (MLC2) (#3672), phospho-MLC2 (Ser19) (#3675), LC3 A/B (#4108), and p-(S)-CDKs Substrate (#9477), obtained from Cell Signaling Technology. Antibodies against GAPDH (#MAB374) were purchased from Millipore. Horseradish peroxidase-conjugated goat anti-mouse or anti-rabbit (Southern Biotechnology) antibodies were then incubated for 1 h and revealed with the SuperSignal West Pico® reagent (Thermo Fisher Scientific) or the ECL^TM^ Prime Western Blotting Detection System (GE Healthcare) using an ImageQuant LAS 4000 software-assisted imager (GE Healthcare).

### Statistical analyses

Each experiment has been repeated at least three times, yielding comparable results. Means and standard errors (SEM) of three independent biological replicates are shown. Data were analyzed by means of Prism v. 5.03 (GraphPad Software, La Jolla, CA, USA). Statistical significance was assessed by one-way analysis of variance tests. In all experiments, *p* values <0.05 were considered as statistically significant.

## Electronic supplementary material

Supplementary Information

Supplementary Figure 1

Supplementary Figure 2

Supplementary Figure 3

Supplementary Figure 4

Supplementary Figure 5

Supplementary Figure 6
