# Supplementary material for: Anticancer chemotherapy and radiotherapy trigger both non-cell-autonomous and cell-autonomous death
Source: Cell Death Dis. 2018 Jun 18;9(7):716. doi: 10.1038/s41419-018-0747-y (PMC6006149; doi:10.1038/s41419-018-0747-y)
Supplement: Supplementary file 1 — Supplementary Information [file 41419_2018_747_MOESM1_ESM.docx]

**Supplementary Information**

Supplementary Information contains 4 supplementary figures.

**Supplementary Figure 1** Validation of pharmacological inhibitors used during cell death profiling. (a) Effect of 30 μM Y27632 on Myosin Light Chain 2 (MLC2) phosphorylation. Human colon carcinoma HCT116^WT^ cells were incubated in presence or absence of 30 μM Y27632 for 24 hours and analyzed for the phosphorylation of Myosin Light Chain 2 on serine 19 (MLC2S19*) and the expression of MLC2 by western blot. GAPDH was used as loading control. Representative western blots of 3 independent experiments are shown. (b) Effect of 100 μM Z-VAD-fmk on caspase-3 cleavage. HCT116 ^WT^ cells were treated with 100 μM of Z-VAD-fmk (ZVAD) alone or combined with 75 μM cisplatin (CDDP) for 24 hours and analyzed for the cleavage of caspase 3 and caspase 3 expression by western blot. GAPDH was used as loading control. Representative western blots of 3 independent experiments are shown. (c) Effect of 50nM bafilomycin A1 on autophagy induction. HCT116 ^WT^ cells were treated with 50nM bafilomycin A1 (BafA1) for 12 hours and analyzed for the conversion of autophagic marker LC3 I into LC3 II. GAPDH was used as loading control. Representative western blots of 3 independent experiments are shown. (d) Effect of roscovitine on the phosphorylation of CDKs. HCT116 ^WT^ cells were treated with 100 nM nocodazole (NCZ) alone or combined with roscovitine (Rosco) at the indicated concentrations for 48 hours. Cells were then analyzed for the phosphorylation of CDKs, p-(S)-CDKs substrate. GAPDH was used as loading control. Representative western blots of 3 independent experiments are shown. (e) Effect of 30 μM NEC1 on necroptosis elicited by TNF+ZVAD treatment. Murine fibrosarcoma cells L929 were treated with Necrostatin-1 (NEC1) and Z-VAD-fmk (ZVAD) for 1 hour and stimulated with TNF (20ng/ml) for 16 hours. The loss of plasma membrane integrity was quantified using propidium iodide (PI) uptake by flow cytometry. Data are presented as mean ± SEM of 3 independent experiments. * is used for the comparison of “TNF- or TNF+ZVAD-treated cells” with “untreated cells”, # for the comparison of “TNF+NEC1-treated cells” with “TNF-treated cells” and & for the comparison of “TNF+ZVAD+NEC1-treated cells” with “TNF+ZVAD-treated cells”. **** or #### or &&&& p<0.0001.

**Supplementary Figure 2** Analysis of γ-irradiation–elicited cell cycle distribution by quantitative imaging flow-cytometry and by classical flow-cytometry. (a-h) Cell cycle analysis (with Hoechst 33342) were performed for total cell population (CMTMR^+^ or CMFDA^+^ HCT116^WT^ (a, b), HCT116^+/+^ cells (c, f) and CMTMR^+^ or CMFDA^+^ MCF7 cells (i, l)), for the untreated (red) CMTMR^+^ HCT116^WT^ cells (a, b), CMTMR^+^ HCT116^+/+^ cells (d, g) and CMTMR^+^ MCF7 cells (j, m), for untreated (green) CMFDA^+^ HCT116 cells (a, b, c, e, f and h) and CMFDA^+^ MCF7 cells (i, k, l, n), and finally for treated (green) CMFDA^+^ HCT116^WT^ cells (b), CMFDA+ HCT116^+/+^ cells (e, h) and CMFDA^+^ MCF7 cells (k, n). Cell cycle distribution was detected after 24-hour (a-e) or 48-hour (f-h) co-culture of untreated (red) CMTMR-labeled HCT116^WT^ cells or HCT116^+/+^ cells with untreated (green) CMFDA-labeled HCT116^WT^ cells or HCT116^+/+^ (a, c and f), and co-culture of untreated (red) CMTMR-labeled HCT116^WT^ cells or HCT116^+/+^ cells with (green) CMFDA-labeled HCT116^WT^ cells or HCT116^+/+^ cells that have been irradiated with indicated doses of γ-ionizing radiation (b, d, e, g and h). Similarly, cell cycle distributions were determined after 12-hour (i-k) or 24-hour (l-n) co-culture of untreated (red) CMTMR-labeled MCF7 cells and untreated (green) CMFDA-labeled MCF7 cells (i, l) or of untreated (red) CMTMR-labeled MCF7 cells with (green) CMFDA-labeled MCF7 cells that have been irradiated with indicated doses of γ-ionizing radiation (j, k, m, n). (o-q) Co-cultures of wild-type (WT) HCT116 (HCT116^WT^) cells were also performed during 24 hours in presence or absence of the indicated pharmacological death effector inhibitors. Cell cycle distributions were determined as previously described for the untreated (red) CMTMR^+^ HCT116^WT^ cells, for untreated (green) CMFDA^+^ HCT116^WT^ cells, for treated (green) CMFDA^+^ HCT116^WT^ cells and for total cell population (CMTMR^+^ or CMFDA^+^ HCT116^WT^ cells). Representative dot plots (a, b) and quantitative data (c-q) are shown (means ± SEM, n = 3). (r, s) Cell cycle analyses (with Hoechst 33342) by classical flow-cytometry were performed after 24-hour co-culture of untreated HCT116^WT^ cells with HCT116 ^WT^ cells that have been irradiated with 4 grays of γ-ionizing radiation or of untreated HCT116 ^WT^ cells with untreated HCT116 ^WT^ cells in presence or absence of the indicated inhibitors. Representative dot plots are shown (r) and quantitative data (s) are reported (means ± SEM, n = 3).

**Supplementary Figure 3** Determination of cell cycle distribution elicited by paclitaxel using a quantitative imaging flow-cytometry. In parallel to the cell death profiling that have been performed in Figure 3, cell cycle analysis (with Hoechst 33342) were performed for total cell population (CMTMR^+^ or CMFDA^+^ HCT116^+/+^ cells (a, d) and CMTMR^+^ or CMFDA^+^ MCF7 cells (g, j)), for the untreated (red) CMTMR^+^ HCT116^+/+^ cells (b, e) and CMTMR^+^ MCF7 cells (h, k), for untreated (green) CMFDA^+^ HCT116^+/+^ cells (a, c, d, and f) and CMFDA^+^ MCF7 cells (h, k), and for treated (green) CMFDA^+^ HCT116^+/+^ cells (c, f) and CMFDA^+^ MCF7 cells (i, l). Cell cycle distribution was detected after 24 hour (a-c) or 48 hour (d-f) co-culture of untreated (red) CMTMR-labeled HCT116^+/+^ cells with untreated (green) CMFDA-labeled HCT116^+/+^ cells (a and d), and co-culture of untreated (red) CMTMR-labeled HCT116^+/+^ cells with (green) CMFDA-labeled HCT116^+/+^ cells that have been treated with indicated concentrations of P (b, c, e and h). Similarly, cell cycle distributions were determined after 12-hour (g-i) or 24-hour (j-l) co-culture of untreated (red) CMTMR-labeled MCF7 cells and untreated (green) CMFDA-labeled MCF7 cells (g, j) or of untreated (red) CMTMR-labeled MCF7 cells with (green) CMFDA-labeled MCF7 cells that have been treated with indicated concentrations of P (h, k, i, l). (m-o) Co-cultures of wild-type (WT) HCT116 (HCT116^WT^) cells were also performed during 24 hours in presence or absence of the indicated pharmacological death effector inhibitors. Cell cycle distributions were determined as previously described for the untreated (red) CMTMR^+^ HCT116^WT^ cells, for untreated (green) CMFDA^+^ HCT116^WT^ cells, for treated (green) CMFDA^+^ HCT116^WT^ cells and for total cell population (CMTMR^+^ or CMFDA^+^ HCT116^WT^ cells). Quantitative data (c-q) are shown (means ± SEM, n = 3).

**Supplementary Figure 4** Determination of cell cycle distribution elicited by oxaliplatin and cisplatin using a quantitative imaging flow-cytometry. In parallel to the cell death profiling that have been performed in Figure 4, cell cycle analysis (with Hoechst 33342) were performed for total cell population (CMTMR^+^ or CMFDA^+^ HCT116^+/+^ cells (a, d) and CMTMR^+^ or CMFDA^+^ MCF7 cells (g, j)), for the untreated (red) CMTMR^+^ HCT116^+/+^ cells (b, e) and CMTMR^+^ MCF7 cells (h, k), for untreated (green) CMFDA^+^ HCT116^+/+^ cells (a, c, d, and f) and CMFDA^+^ MCF7 cells (h, k), and finally for treated (green) CMFDA^+^ HCT116^+/+^ cells (c, f) and CMFDA^+^ MCF7 cells (i, l). Cell cycle distribution was detected after 24 hour (a-c) co-culture of untreated (red) CMTMR-labeled HCT116^+/+^ cells with untreated (green) CMFDA-labeled HCT116^+/+^ cells (a), and co-culture of untreated (red) CMTMR-labeled HCT116^+/+^ cells with (green) CMFDA-labeled HCT116^+/+^ cells that have been treated with indicated concentrations of P (b and c). Similarly, cell cycle distributions were determined after 12-hour (d-f) or 24-hour (g-i) co-culture of untreated (red) CMTMR-labeled MCF7 cells and untreated (green) CMFDA-labeled MCF7 cells (d, g) or of untreated (red) CMTMR-labeled MCF7 cells with (green) CMFDA-labeled MCF7 cells that have been treated with indicated concentrations of P (e, f, h and i). (m-o) Co-cultures of wild-type (WT) HCT116 (HCT116^WT^) cells were also performed during 24 hours in presence or absence of the indicated pharmacological death effector inhibitors. Cell cycle distributions were determined as previously described for the untreated (red) CMTMR^+^ HCT116^WT^ cells, for untreated (green) CMFDA^+^ HCT116^WT^ cells, for treated (green) CMFDA^+^ HCT116^WT^ cells and for total cell population (CMTMR^+^ or CMFDA^+^ HCT116^WT^ cells). Quantitative data (j-l) are shown (means ± SEM, n = 3).

**Supplementary Figure 5** Detection of cannibal cell death using a quantitative imaging flow-cytometry. (a, b) In parallel to Figures 5d (a) and 6f (b), cells have been sequentially labeled after co-cultures with BV786-conjugated AnnexinV (AV), DRAQ7 (D7) and Hoechst 33342 for the detection of cannibal cell death elicited by IR (a) or PCT (b). (c) In parallel to Figure 6l, cells have been sequentially labeled after co-cultures with BV786-conjugated AnnexinV, DRAQ7 and Hoechst 33342 for the detection of cannibal cell death elicited by OXA and CDDP. Frequencies of cannibal cell death as revealed by the detection of AV^+^DQ7^-^ and DQ7^+^ cannibal cells have been determined and shown (means ± SEM, n = 3). For supplementary Figures 5b and 5c, * is used for the comparison of “HCT116^WT^ +PCT-treated control (Co.) HCT116^WT^ cells” with “HCT116^WT^ + control (Co.) HCT116^WT^ cells” for AV^+^D7^-^, # for the comparison of “inhibitor-treated cells” with respective “control cells” for AV^+^D7^-^ and δ for the comparison of “inhibitor-treated cells” with respective “control cells” for D7^+^. * or # or & represents p<0.05, ** or ## or $$ or && p<0.01, *** or ### or &&& p<0.001 and **** or &&&& p<0.0001.

**Supplementary Figure 6** Determination of cell cycle distribution of HCT116 ^p53R248W/+^ cells elicited by oxaliplatin, cisplatin, paclitaxel or γ-irradiation using a quantitative imaging flow-cytometry. In parallel to the analyses of the cell death profiling elicited after 24-hour (a, c, e) or 48-hour (b, d, f) co-cultures of untreated (red) CMTMR-labeled p53^R248W/+^ HCT116 cells with untreated (green) CMFDA-labeled p53^R248W/+^ HCT116 cells, with OXA-, CDDP-, PACLI- or IR-treated (green) CMFDA-labeled p53^R248W/+^ HCT116 cells, cell cycle distributions of untreated (red) CMTMR^+^ p53^R248W/+^ HCT116 cells (c, d), untreated (green) CMFDA^+^ HCT116 ^R248W/+^ cells (e, f), treated (green) CMFDA^+^ HCT116 ^R248W/+^ cells (e, f) and on total cell population (as revealed by CMTMR^+^ or CMFDA^+^ HCT116 ^R248/W^ cells (a, b) (means ± SEM, n = 3).
