# Supplementary figures and images for: Anticancer chemotherapy and radiotherapy trigger both non-cell-autonomous and cell-autonomous death
Source: Cell Death Dis. 2018 Jun 18;9(7):716. doi: 10.1038/s41419-018-0747-y (PMC6006149; doi:10.1038/s41419-018-0747-y)

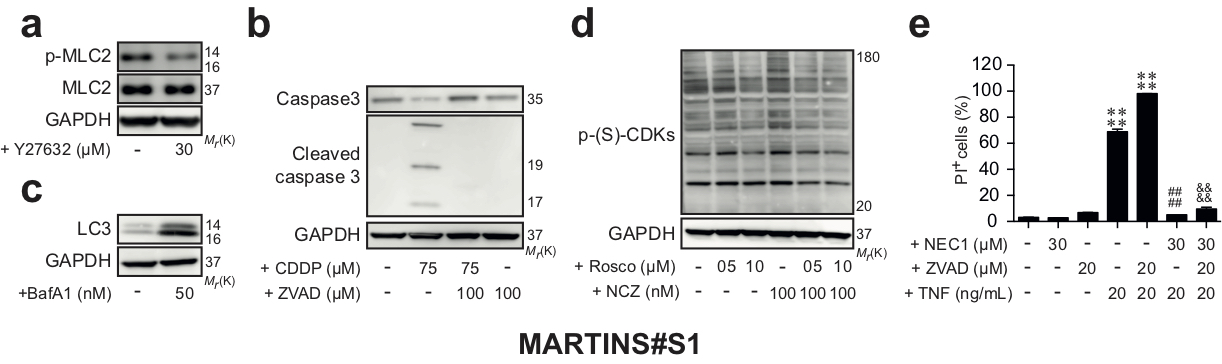

Supplement: Supplementary file 2 — Supplementary Figure 1 [file 41419_2018_747_MOESM2_ESM.jpg]

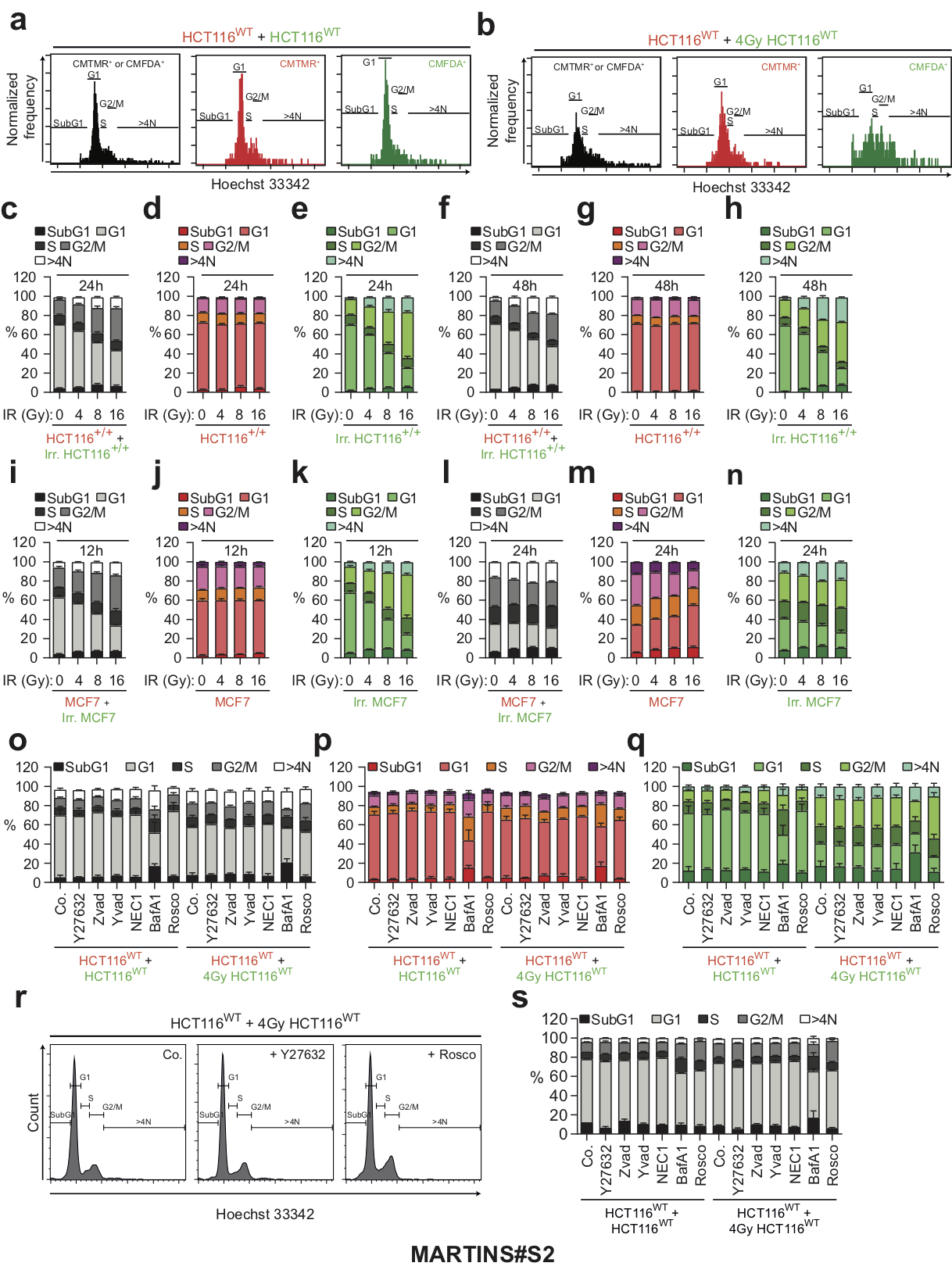

Supplement: Supplementary file 3 — Supplementary Figure 2 [file 41419_2018_747_MOESM3_ESM.jpg]

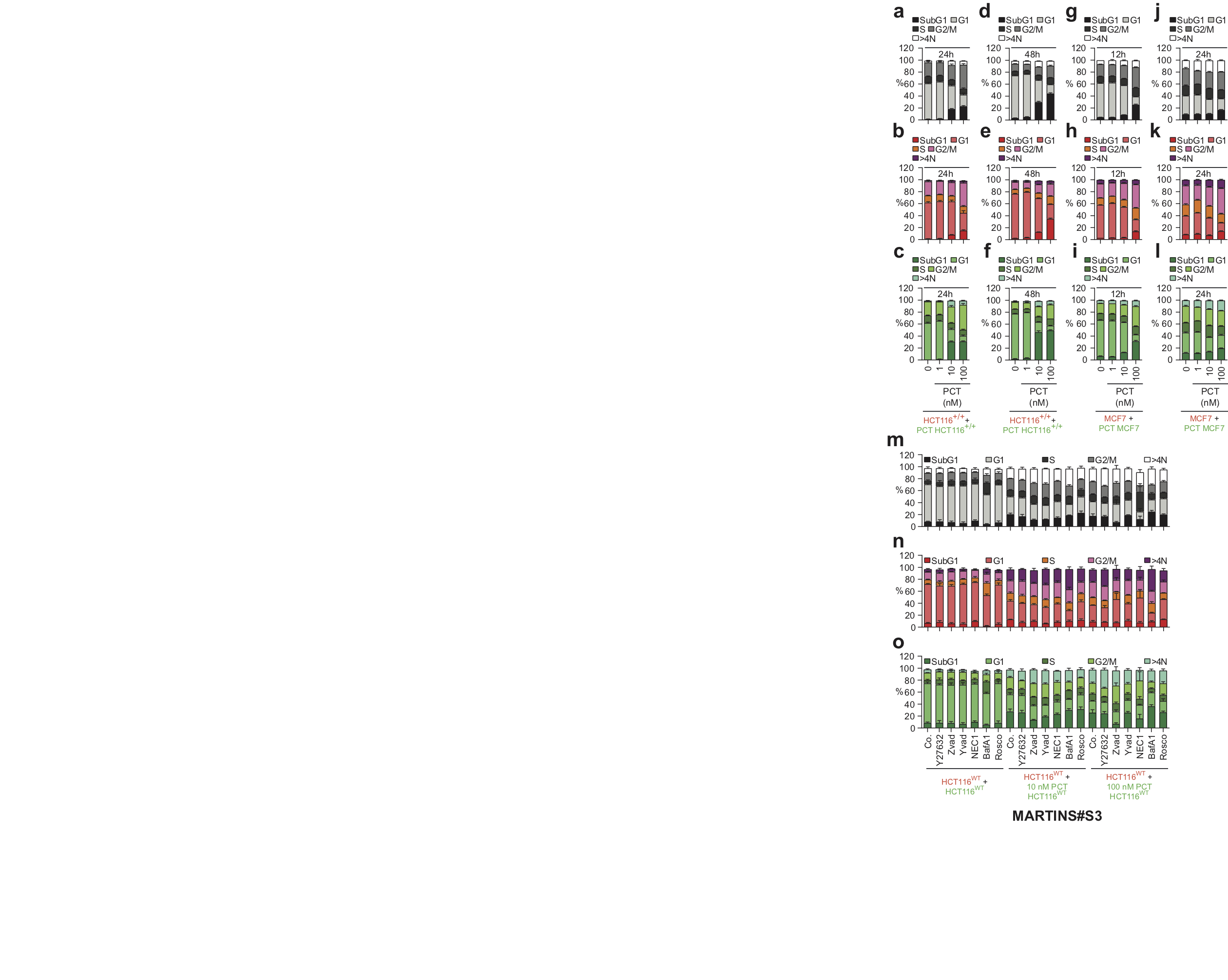

Supplement: Supplementary file 4 — Supplementary Figure 3 [file 41419_2018_747_MOESM4_ESM.jpg]

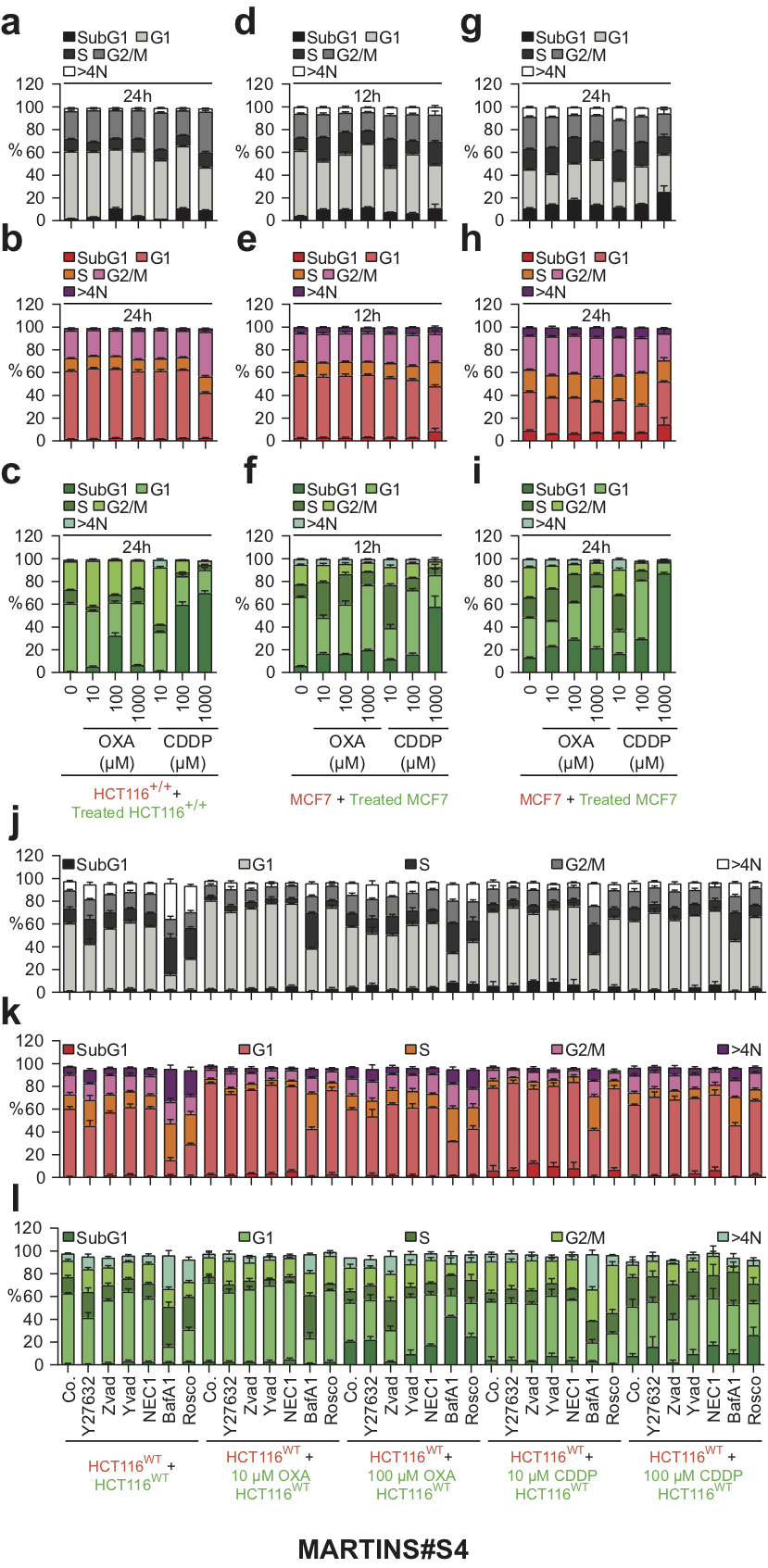

Supplement: Supplementary file 5 — Supplementary Figure 4 [file 41419_2018_747_MOESM5_ESM.jpg]

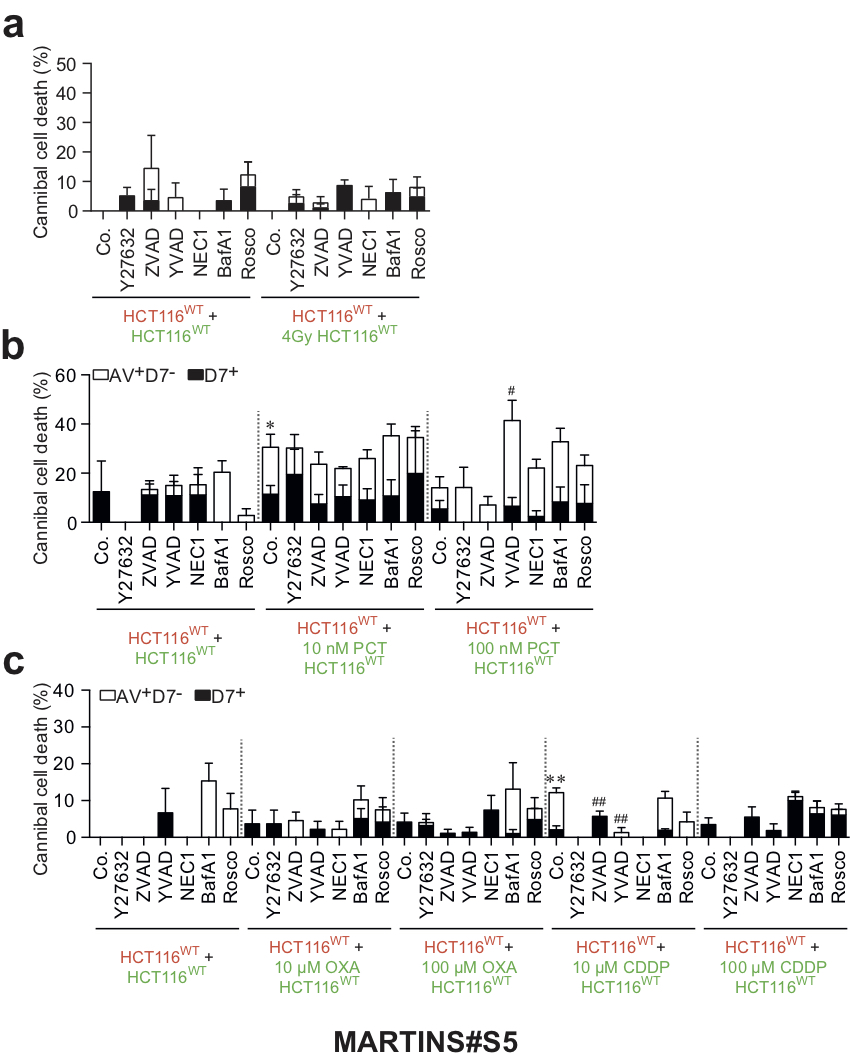

Supplement: Supplementary file 6 — Supplementary Figure 5 [file 41419_2018_747_MOESM6_ESM.jpg]

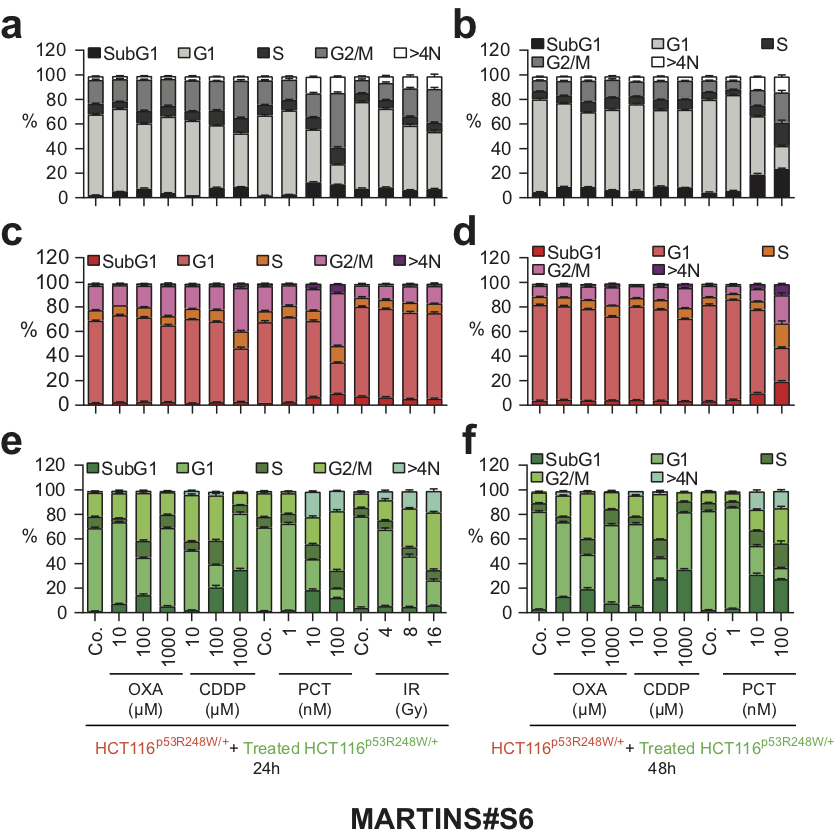

Supplement: Supplementary file 7 — Supplementary Figure 6 [file 41419_2018_747_MOESM7_ESM.jpg]
